# Immediate Effects of Transcutaneous Spinal Cord Stimulation on Motor Function in Chronic, Sensorimotor Incomplete Spinal Cord Injury

**DOI:** 10.3390/jcm9113541

**Published:** 2020-11-02

**Authors:** Christian Meyer, Ursula S. Hofstoetter, Michèle Hubli, Roushanak H. Hassani, Carmen Rinaldo, Armin Curt, Marc Bolliger

**Affiliations:** 1 Spinal Cord Injury Center, Balgrist University Hospital, Forchstrasse 340, 8008 Zurich, Switzerland; christian.meyer@balgrist.ch (C.M.); Michele.Hubli@balgrist.ch (M.H.); Roushanak.Hassani@balgrist.ch (R.H.H.); carmen_rinaldo@hotmail.com (C.R.); Armin.Curt@balgrist.ch (A.C.); marc.bolliger@balgrist.ch (M.B.); 2 Center for Medical Physics and Biomedical Engineering, Medical University of Vienna, 1090 Vienna, Austria

**Keywords:** human, non-invasive, spinal cord injury, spinal cord stimulation, spinal reflexes, voluntary ankle control, walking

## Abstract

Deficient ankle control after incomplete spinal cord injury (iSCI) often accentuates walking impairments. Transcutaneous electrical spinal cord stimulation (tSCS) has been shown to augment locomotor activity after iSCI, presumably due to modulation of spinal excitability. However, the effects of possible excitability modulations induced by tSCS on ankle control have not yet been assessed. This study investigated the immediate (i.e., without training) effects during single-sessions of tonic tSCS on ankle control, spinal excitability, and locomotion in ten individuals with chronic, sensorimotor iSCI (American Spinal Injury Association Impairment Scale D). Participants performed rhythmic ankle movements (dorsi- and plantar flexion) at a given rate, and irregular ankle movements following a predetermined trajectory with and without tonic tSCS at 15 Hz, 30 Hz, and 50 Hz. In a subgroup of eight participants, the effects of tSCS on assisted over-ground walking were studied. Furthermore, the activity of a polysynaptic spinal reflex, associated with spinal locomotor networks, was investigated to study the effect of the stimulation on the dedicated spinal circuitry associated with locomotor function. Tonic tSCS at 30 Hz immediately improved maximum dorsiflexion by +4.6° ± 0.9° in the more affected lower limb during the rhythmic ankle movement task, resulting in an increase of +2.9° ± 0.9° in active range of motion. Coordination of ankle movements, assessed by the ability to perform rhythmic ankle movements at a given target rate and to perform irregular movements according to a trajectory, was unchanged during stimulation. tSCS at 30 Hz modulated spinal reflex activity, reflected by a significant suppression of pathological activity specific to SCI in the assessed polysynaptic spinal reflex. During walking, there was no statistical group effect of tSCS. In the subgroup of eight assessed participants, the three with the lowest as well as the one with the highest walking function scores showed positive stimulation effects, including increased maximum walking speed, or more continuous and faster stepping at a self-selected speed. Future studies need to investigate if multiple applications and individual optimization of the stimulation parameters can increase the effects of tSCS, and if the technique can improve the outcome of locomotor rehabilitation after iSCI.

## 1. Introduction

Supraspinal input to spinal circuits is critical for the initiation and control of human locomotion [1,2]. In particular, the activation of ankle dorsiflexors in the swing phase is strongly related to corticospinal transmission [3,4]. After an incomplete spinal cord injury (iSCI), reduced corticospinal input causes muscle weakness and spasticity, which impair voluntary ankle control [5,6,7]. Deficient ankle control after iSCI often accentuates walking impairments and poses a challenge to regaining walking function [8,9]. Assisting locomotor recovery after iSCI and improving its outcome is of high relevance for affected individuals, and would considerably increase their quality of life [10,11].

Intensive training, in combination with electrical stimulation of the lumbar spinal cord using epidurally implanted electrodes, has been shown to improve, or induce regains of, voluntary control over lower limb movements and over-ground walking capabilities in individuals with chronic, severe SCI [12,13,14,15,16]. Epidural spinal cord stimulation (eSCS) activates afferent fibers in the posterior roots, and generates multisegmental input to spinal networks [17,18,19,20,21]. Previous studies have demonstrated a variety of immediate (i.e., without training), frequency-dependent effects of eSCS on lower limb motor control after SCI. Specifically, eSCS in the range of 5–15 Hz was shown to induce bilateral lower limb extension and upright standing within single application sessions [15,22]. At frequencies around 30 Hz, eSCS immediately generated rhythmic flexion-extension movements of the lower limbs of individuals with motor complete SCI lying supine [21,23,24], allowed enhanced rhythmic lower limb muscle activities induced by proprioceptive input generated during assisted treadmill stepping [15,25], and enabled voluntary hip and knee flexion and extension [15], as well as ankle dorsiflexion [16]. Stimulation at frequencies ≥50 Hz was associated with the attenuation of lower-limb spasticity [26]. However, eSCS is an invasive procedure, with the corresponding risks for treated patients. The same neural input structures to the spinal cord can be less specifically, but non-invasively, stimulated with surface electrodes [27,28,29]. Similarly to eSCS, transcutaneous spinal cord stimulation (tSCS) has been shown to immediately induce lower limb extension at 15 Hz [30,31] and to attenuate spasticity at 50 Hz [32,33,34]. Furthermore, initiation of flexion–extension movements and an immediate augmentation of locomotor activity was also reported for 30-Hz tSCS [35,36,37]. No study so far has compared the impact of different stimulation frequencies on the augmentation of residual voluntary motor control. Specifically, the effects of tSCS on more finely controlled ankle movements, which could be an origin of improvements in locomotor function, has not yet been assessed. Several mechanisms were suggested to be responsible for the modulatory effects of electrical lumbar SCS, including an increase in excitability of spinal locomotor networks [16,38,39] and the regulation of activity in multiple segmental circuits [34,40]. However, the recruitment of specific spinal circuits, also dependent on the different stimulation frequencies applied, has not yet been shown.

In this study, we investigated the immediate effects of a single application of tSCS at three different frequencies (15 Hz, 30 Hz, and 50 Hz) on voluntary ankle control, studied in the supine position in ten individuals with chronic, sensorimotor iSCI. We hypothesized that tSCS would immediately facilitate ankle control similar to eSCS, and that this facilitation would be strongest at 30 Hz [16,21], i.e., at a frequency previously shown for eSCS to promote rhythmic flexion-extension movements Moreover, we investigated whether the effects observed in the ankle control task would transfer to over-ground walking with a body weight support (BWS) system and if other stimulation-induced effects were present during locomotion.

Besides effects on motor control, this is the first study to explore the effect of tSCS on dedicated spinal networks by the electromyographic (EMG) representation of a polysynaptic spinal reflex elicited by electrical stimulation of the distal tibial nerve, hereinafter termed spinal reflex. Previous studies have suggested an association of this reflex with spinal locomotor circuits and their functional state after SCI [41,42,43]. In neurologically intact individuals, the spinal reflex is characterized by a dominant early EMG component occurring at latencies of 60–120 ms, while in individuals with severe SCI, an additional late EMG component at latencies of 120–450 ms gradually develops in the weeks post-injury [41,42,44,45]. Dominant late spinal reflex components were associated with more impaired locomotor function [42]. We hypothesized that afferent input generated by 30-Hz tSCS would interact with the spinal locomotor circuits, resulting in facilitated early, and diminished late reflex components under tonic stimulation.

## 2. Materials and Methods

### 2.1. Participants

Data were derived from ten individuals with chronic, sensorimotor iSCI, classified as grade D on the American Spinal Injury Association Impairment Scale [46]. Details on the neurological status of the participants and the assistance required for walking are provided in Table 1. Lower extremity motor scores and sensory scores were taken from previous clinical assessments, and the walking index for spinal cord injury (WISCI II) [47] was assessed in this study. Participant 3 had additional lesions affecting the efferent system associated with his left lower limb. None of the participants had previous experience with tSCS. Among the exclusion criteria were neurological lesion levels caudal to T12, and active or passive implants at vertebral level T9 or caudally, such as osteosynthesis material. The study was approved by the cantonal ethics committee of Zurich, Switzerland (KEK-ZH 2017-00053), and conducted in accordance with the Declaration of Helsinki. All participants signed written informed consent prior to their enrollment into the study.

### 2.2. Study Protocol

Two independent study sessions were conducted on separate days (time between sessions: 1–21 days, same order of sessions for all participants). In session 1, the participants’ ability to perform controlled ankle movements was investigated. In session 2, spinal reflex activity and walking performance were assessed.

For the ankle control assessment, participants were lying supine with their thighs and shanks stabilized by a vacuum pillow without restricting the full range of motion (ROM) of the ankle joints. Participants were able to see their feet during the tests. The assessment comprised two protocols. First, participants performed 25 cycles of unilateral rhythmic ankle movements, from maximum dorsi- to maximum plantar flexion angles, at a target rate given by an auditory cue. Three different movement rates were tested, in this sequence, 0.8 Hz, 1.6 Hz, and 2.4 Hz [49]. Second, participants performed a unilateral irregular precision task. It consisted of unilateral ankle movements following a predefined, irregular, sinusoidal trajectory projected on a screen. A motion capture system (Vicon Motion Systems Ltd., Oxford, UK) provided visual feedback of the executed ankle movements in relation to the targeted trajectory. The maxima of the trajectory were adjusted to 90% of the individual maximum ankle ROM. Each protocol was repeated five times (first right side and then left side per repetition), first without tSCS (tSCS-off), followed by three repetitions under tonic tSCS at 15 Hz, 30 Hz, and 50 Hz (applied in a randomized order), and finally without tSCS again (tSCS-off_rep_). Between repetitions, participants rested as needed.

Spinal reflexes were elicited by applying monopolar electrical stimulation to the distal tibial nerve at the dorsal aspect of the medial malleolus (anode and cathode over the nerve with minimal distance but without contact) through self-adhesive hydrogel surface electrodes (Neuroline 700, Ambu, Ballerup, Denmark) with participants in the supine position (knees supported with a cushion). A constant current stimulator (Dantec Keypoint Focus Workstation, Natus Medical Incorporated, Pleasanton, CA, USA) was set to deliver trains of five (five participants) or eight monophasic rectangular pulses, each with a duration of 1.0 ms and an interpulse interval of 4 ms (250 Hz). The total time of a stimulus was 17 ms (five pulses) or 29 ms (eight pulses). Stimulation amplitude was gradually increased in 1-mA increments from 1 mA up to the reflex threshold (spinal reflexes with amplitudes > 20 µV in three out of five stimuli). Subsequently, five stimuli with an amplitude of 1.5 times the reflex threshold were applied. The protocol was conducted first without and then under tonic 30-Hz tSCS on the right limb, and subsequently in the same order on the left limb. For the five above-threshold stimuli, the same stimulation amplitudes were used in the tSCS-off and tSCS-on conditions (1.5 times the reflex threshold of tSCS-off). To minimize habituation, stimulation trains were delivered at randomized intervals of 30–40 s [50], and participants were given a backward counting task [51,52]. The positioning of the participants and the limb was kept constant between the two conditions.

For the gait assessments, participants were fitted with a harness to the BWS system (The FLOAT, Reha-Stim Medtec AG, Schlieren, Switzerland) [48,53,54], set to the individual minimum that allowed unrestricted over-ground walking over a distance of 7 m (Table 1; same BWS provided for tSCS-off and tSCS-on conditions), but always ≥4 kg (fall detection limit). Participants 1, 7, and 8 additionally required a walker, and participant 4 used two crutches. Walk tests comprised the assessment (i) of the minimum time required to walk 7 m without tSCS, as well as under tonic 30-Hz tSCS, used to calculate the maximum walking speed (m/s); and (ii) of gait kinematics and lower limb muscle activity without (first condition) tSCS, and during tSCS at 15 Hz, 30 Hz, and 50 Hz (applied in a randomized sequence) while walking 7 m at a self-selected speed. To avoid confounding effects, trials under tonic tSCS were repeated if self-selected walking speed deviated by more than ±10% from that of the tSCS-off condition. Two trials of each assessment were conducted per condition. Participants 1–6 completed both assessment parts and fulfilled the criterion of constant walking speed in the self-selected walking condition (four trials repeated). Participants 7 and 8 showed signs of fatigue during walking, completed the 7 m with major fluctuation of walking speed across all tSCS-off and tSCS-on trials, and were unable to fulfill the criterion of constant walking speed across repetitions. In both participants, the maximum walking speed was not assessed, and self-selected walking speed under different tSCS conditions are reported instead. Walk tests were not conducted in participants 9 and 10 since no posterior root-muscle (PRM) reflexes [27,28] were elicitable in the BWS- and walker-assisted standing position (see below).

### 2.3. EMG and Kinematic Data

EMG activity was recorded from bilateral rectus femoris (RF), vastus medialis (VM), semitendinosus (ST), tibialis anterior (TA), and medial gastrocnemius (MG) with surface electrodes (Kendall H124SG, Covidien, Medtronic, Dublin, Ireland), placed in accordance with the European recommendations for surface electromyography (SENIAM [55]), and using the Aktos system (myon AG, Schwarzenberg, Switzerland). Data were amplified with a gain of 1000 over a bandwidth of 10–500 Hz and digitized at 2000 samples per second and channel. For the spinal reflex protocol, the rectified EMG activity of bilateral TA was recorded using surface electrodes (BlueSensor NF, Ambu, Ballerup, Denmark), and a Dantec Keypoint Focus Workstation over a bandwidth of 20 Hz to 3000 Hz and digitized at 6000 samples per second. A copper strip covered in a wet absorbent felt placed around the ankle joint was used as ground electrode.

Kinematic data were acquired using the Vicon motion capture system (Vicon Motion Systems Ltd., Oxford, UK). For the ankle control assessment, three reflective markers were placed bilaterally on the fibula head, the lateral malleolus, and the fifth metatarsal head. For the walk tests, a total of 42 markers were placed according to the plug-in gait model (Vicon Motion Systems Ltd., Oxford, UK), with additional locations for gap filling purposes. Kinematic data were sampled at 100 Hz (ankle control assessment) or 200 Hz (walk tests), labelled and gap filled using Vicon Nexus 2.6, and synchronized to the EMG data (without upscaling). Trajectories of the walk tests were additionally filtered (Woltring filter, volume specific mean-square-error value of 15) and modelled with the plug-in gait model [56]. Gait events were manually detected based on marker position data. Angular excursions of the lower limb joints were calculated based on vectors using toe, ankle, knee, and sacrum markers, as well as the modelled hip joint rotation center [57,58].

### 2.4. Transcutaneous Spinal Cord Stimulation

Lumbar tSCS was applied through a self-adhesive surface electrode (5 × 9 cm; RehaTrode, Hasomed GmbH, Magdeburg, Germany) positioned longitudinally over the spine, covering the T11 and T12 spinous processes, and a coupled pair of electrodes (7.5 × 13 cm each) placed over the lower abdomen, in symmetry to the umbilicus [27,28,59]. A current-controlled stimulator (RehaMove 3.0, Hasomed GmbH) was set to deliver charge-balanced, symmetric, biphasic rectangular pulses of 1 ms width per phase. With reference to the abdominal electrodes, the paraspinal electrode was the anode for the first, and the cathode for the second, pulse phase [28]. Paravertebral electrode position over the lumbar and upper sacral spinal cord was verified by the elicitation of PRM reflexes in the L2–S2 innervated RF, VM, ST, TA, and MG bilaterally [27,28]. Stimulation of afferent fibers was tested using a paired-pulse paradigm with interstimulus intervals of 30 ms, 50 ms, and 120 ms [27,28,60,61,62,63,64]. In session 1, the mean PRM-reflex threshold ± SD across participants was 31.0 ± 18.8 mA (per phase of the biphasic stimulation pulse), ranging from 15 mA to 70 mA. Tonic tSCS was applied with a target amplitude of 0.8–1.0 times the individual PRM-reflex threshold [16,36], yet always below a level causing discomfort, and which amounted to 26.4 ± 17.3 mA across participants (same for all stimulation frequencies, 13–65 mA; 0.84 ± 0.10 × PRM-reflex threshold). In session 2, the mean PRM-reflex threshold was 32.0 ± 14.4 mA (14–65 mA) in the supine position (spinal reflex protocol), and 40.6 ± 13.5 mA (20–60 mA) in the BWS-supported standing position with walking aids, as used for stepping. The mean stimulation amplitude of tonic tSCS was 28.8 ± 14.7 mA (13–65 mA, 0.89 ± 0.08 × PRM reflex threshold) for the spinal reflex protocol, and 34.8 ± 13.1 mA (same for all stimulation frequencies, 16–60 mA, 0.85 ± 0.10 × PRM reflex threshold) for the walk tests. The maximum durations of tSCS application with the target amplitude were approximately 5 min for the ankle control and walk tests, and 15 min for the spinal reflex assessment. Participants 4, 5, 8, and 10 reported paresthesia in lower limb dermatomes during the tonic stimulation [34,65].

### 2.5. Data Analysis and Statistics

Data were analyzed offline using Matlab 2017b (The Mathworks Inc., Natick, MA, USA), IBM SPSS Statistics 26.0 for Windows (IBM Corporation, Armonk, NY, USA), and R 3.6.1 (The R project for Statistical Computing, R Core Team) with R Studio 1.2 (R Studio, Inc., Boston, MA, USA). α-errors of *p* < 0.05 were considered significant. Prior to the data analysis, the two legs of each participant were grouped into more and less affected sides, based on the total lower extremity motor scores (LEMS) per leg [66,67,68]. In participant 7, total light touch sensory scores were additionally considered, as LEMS were equivalent in both legs. This separation was applied to account for asymmetrical damages in SCI, to make legs within a group more comparable, and to allow inferences on impairment level.

For the unilateral rhythmic ankle dorsi- and plantar flexion movement, ankle ROMs, root-mean-square (RMS) values of the EMG signals of TA during dorsiflexion, and MG during plantar flexion, as well as deviations from the target movement rate, were determined for 20 movement cycles of each repetition of the task (the first five of the 25 cycles per repetition were omitted) and averaged. Separate linear mixed models (due to several factors and missing values), with movement rate (0.8 Hz, 1.6 Hz, and 2.4 Hz) and tSCS condition (tSCS-off, 15-Hz tSCS, 30 Hz tSCS, 50-Hz tSCS, tSCS-off_rep_) as fixed effects and between-subject differences as random effect, were fit to the data of the more and the less affected lower limbs. Residuals of linear mixed models were visually screened for normal distribution (QQ-plots), and input datasets were transformed if necessary (log or square root transformations). Post-hoc tests were based on estimated marginal means and Bonferroni-corrected to adjust for multiple comparisons. Effect sizes are reported by the partial eta-squared ($\eta_{p}^{2}$). Participant 4 did not perform the tSCS-off_rep_ condition (fatigue) and participant 2 was unable to perform the highest movement rate with his more affected lower limb. For the irregular ankle movements, deviations from the predefined target trajectory were calculated as the RMS-error normalized to the total range of the trajectory (90% of the individual maximum ankle ROM) and compared using linear mixed models. In participants 4 and 8, the irregular ankle movement test was not conducted due to fatigue. Across ankle control assessments, data derived from the less affected lower limb of participant 1 were considered only (additional lesions affecting efferent system in more affected limb), and the more affected leg of participant 1 was excluded from analysis because of 0° ankle ROM in all conditions.

In the recordings of session 2, stimulation artifacts produced by tSCS were superimposed on the EMG activities, generated in TA during the spinal reflex protocol, and on those of the thigh muscles during the walk tests. Prior to further analysis, the artefacts were removed offline by adjustable blanking intervals of 3–6 ms, beginning with the leading edge of the stimulus and covering any die-away effects of the falling stimulus edge [36] (cf. Figure 1 in [36] for illustration). Within the blanking intervals, EMG traces were set to not-a-number. In the spinal reflex protocol, the same procedure was applied to the EMG recordings in the tSCS-off condition for an assumed 30-Hz stimulation train to avoid confounding effects on the evaluation of the early and late reflex components (see below). For the walk tests, where only increased EMG activity was expected during stimulation, the procedure was applied to the tSCS-on conditions only, thus rather under- than overestimating the EMG activity of the thigh muscles generated in the trials with tonic stimulation.

Spinal reflexes obtained with above-threshold stimulation were analyzed for the occurrence of early and late components [41,42]. Time windows were set at 60–120 ms for the detection of the early component, and at 120–450 ms for the late component [41]. Maximum EMG amplitudes within these windows were determined, and noise-corrected RMS values (a period of noise was manually selected for each recording) of the EMG within ±25 ms of the maxima were calculated. Values of 0 were assigned if no response was detectable within a given time window (i.e., RMS noise ≥ RMS reflex). EMG-RMS values of the early and the late response components, as well as spinal reflex thresholds, were compared by running separate linear mixed models (because more than one factor) with stimulation condition (tSCS-off, 30-Hz tSCS) and lower limb (more affected, less affected) as fixed effects and between-subject differences as random effect (see above for further methodological details of applied linear mixed models). Data derived from the less affected lower limb of participant 3 were excluded from analysis due to the regular occurrence of non-stimulation related muscle twitches in TA that were superimposed on the EMG and impeded reflex threshold estimation. No spinal reflexes were recorded from the less affected lower limb of participant 4 because of the perception of discomfort at the stimulation amplitude required for their elicitation.

Maximum walking speed was compared between tSCS-off and tSCS-on at 30 Hz using a Wilcoxon signed-rank test (due to the limited sample size of 6 participants, correlation coefficient as effect size). For the analysis of gait kinematics at a self-selected speed, mean hip, knee, and ankle ROMs, as well as relative durations of double limb support phases, step lengths, and stride times were derived for the more and less affected lower limbs, separately for the different tSCS-off and tSCS-on conditions. Lower limb muscle activity was characterized by mean EMG-RMS values per muscle, and separately for stance and swing phases. Each outcome was statistically compared between conditions in participants 1–6 (constant self-selected speed) using a Friedman test (due to limited sample size, Bonferroni-corrected for more and less affected leg). Additionally, the results of the walk tests were reported descriptively. For this purpose, data obtained under tonic stimulation were normalized to the respective values in the tSCS-off condition using z-scores (changes in standard deviations from baseline). Given the high kinematic fluctuations in participants 7 and 8, the variability of double limb support phases, step lengths, and stride times was additionally calculated as the coefficient of variation (SD divided by the mean).

For the ankle control tasks, as well as the walk tests, participants were asked to rate their comfort and performance perception in the tSCS-off and tSCS-on conditions (all frequencies combined) based on the visual analogue scale (VAS; 0–100, 0 being the lowest subjective score and 100 being the highest subjective score). Data were not normally distributed (Shapiro–Wilk test) and on-off conditions were consequently compared using Wilcoxon signed-rank tests (correlation coefficient as effect size).

## 3. Results

### 3.1. Voluntary Ankle Control

**Figure 1:**
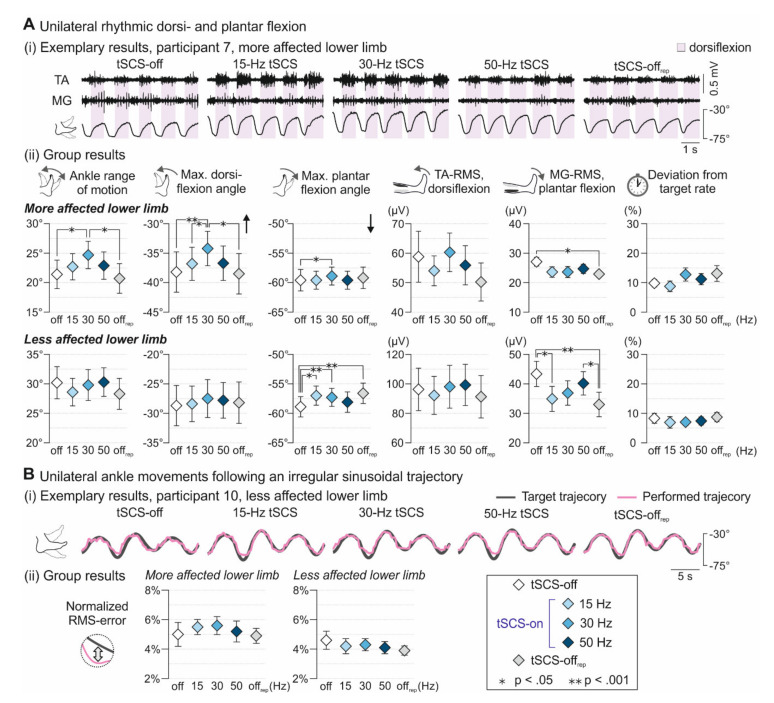
**Effect of tSCS on voluntary unilateral ankle control.** (**A**) Unilateral rhythmic dorsi- and plantar flexion movements. (**i**) EMG activities of tibialis anterior (TA) and medial gastrocnemius (MG) shown for five movement cycles at a rate of 1.6 Hz during tSCS-off, tSCS at stimulation frequencies as indicated, and tSCS-off_rep_ for the more affected lower limb of participant 7. Shaded backgrounds mark dorsiflexion phases, identified based on kinematic data. Under tonic tSCS, maximum ankle angles and TA activity during dorsiflexion were increased, while clonus-like activity in MG that was present in the tSCS-off condition was visibly reduced. (**ii**) Group results (mean ± SE) across movement rates of ankle range of movement; maximum dorsiflexion angles (black arrow indicates direction of dorsiflexion, zero reference represents an absolute joint angle of 90°, i.e., as during an upright standing position); maximum plantar flexion angles (black arrow indicates direction of plantar flexion); TA EMG-RMS values during dorsiflexion; MG EMG-RMS values during plantar flexion; and deviation from target movement rate during tSCS conditions as indicated, separately shown for the more and the less affected lower limbs. (**B**) Unilateral ankle movements following an irregular sinusoidal target trajectory. (**i**) Exemplary recordings of performed ankle movements (purple line) relative to the predefined irregular target trajectory (grey line); less affected lower limb, participant 10. (**ii**) Group results (mean ± SE) of the normalized RMS-error, providing information on the deviation of the performed movement from the target trajectory, across tSCS conditions, shown for the more and the less affected lower limb. Asterisks mark significant results of post-hoc pairwise comparisons. deg., degree; EMG, electromyographic; MG, medial gastrocnemius; RMS, root mean square; TA, tibialis anterior; * *p* < 0.05; ** *p* < 0.001.

Tonic tSCS applied for a few minutes had several immediate effects on voluntary alternating ankle flexion and extension at three different movement rates (Figure 1A). In the more affected lower limb, tSCS condition (tSCS-off, 15-Hz tSCS, 30 Hz tSCS, 50-Hz tSCS, tSCS-off_rep_) had a significant main effect on ankle ROM (F_4;89.016_ = 4.368, *p* = 0.003, $\eta_{p}^{2}$ = 0.164), maximum dorsiflexion (F_4;89.011_ = 6.779, *p* < 0.001, $\eta_{p}^{2}$ = 0.234) as well as plantar flexion angles (F_4;89.005_ = 3.557, *p* = 0.010, $\eta_{p}^{2}$ = 0.138), EMG activity of MG during the plantar flexion phases (F_4;88.037_ = 3.290, *p* = 0.015, $\eta_{p}^{2}$ = 0.130), and on the deviation from the target movement rate (F_4;89.028_ = 2.872, *p* = 0.027, $\eta_{p}^{2}$ = 0.114). Post-hoc pairwise comparisons revealed significantly larger ankle ROM during 30-Hz tSCS than tSCS-off (absolute mean difference: 2.9° ± 0.9°, *p* = 0.013) and tSCS-off_rep_ (3.4° ± 0.9°, *p* = 0.003). Additionally, dorsiflexion angles during 30-Hz tSCS were significantly increased by 4.6° ± 0.9° compared to tSCS-off (*p* < 0.001), by 2.7° ± 0.9° compared to 15-Hz tSCS (*p* = 0.041), and by 3.8° ± 1.0° compared to tSCS-off_rep_ (*p* = 0.002). At the same time, absolute plantar flexion angles were smaller by 1.7° ± 0.5°during 30-Hz tSCS than tSCS-off (*p* = 0.008). MG-RMS values during plantar flexion were reduced in tSCS-off_rep_ compared to tSCS-off (5.7 ± 1.6 µV, *p* = 0.007). In the less affected lower limb, tSCS condition had a significant main effect on ankle ROM (F_4;123.011_ = 2.522, *p* = 0.044, $\eta_{p}^{2}$ = 0.076), plantar flexion angles (F_4;123.003_ = 8.125, *p* < 0.001, $\eta_{p}^{2}$ = 0.290), as well as on TA activity during dorsiflexion (F_4;123.005_ = 2.673, *p* = 0.035, $\eta_{p}^{2}$ = 0.080) and MG activity during plantar flexion (F_4;123.017_ = 8.127, *p* < 0.001, $\eta_{p}^{2}$ = 0.209). Post-hoc pairwise comparisons demonstrated significantly larger absolute plantar flexion angles during tSCS-off than during 15-Hz tSCS (1.9° ± 0.4°, *p* < 0.001), 30-Hz tSCS (1.5° ± 0.4°, *p* = 0.001), and tSCS-off_rep_ (1.8° ± 0.4°, *p* < 0.001). MG-RMS values during plantar flexion were larger during tSCS-off than during 15-Hz tSCS (8.5 ± 2.4 µV, *p* = 0.005) and tSCS-off_rep_ (12.9 ± 2.5 µV, *p* < 0.001) as well as larger during 50-Hz tSCS than during tSCS-off_rep_ (9.8 ± 2.5 µV, *p* = 0.001). No significant interaction between tSCS condition and movement rate existed for any outcome measure derived from the more and less affected lower limbs.

tSCS did not modify the ability to follow a predefined, irregular sinusoidal trajectory (Figure 1B) as quantified by the normalized RMS-error, neither in the more (F_4;17.259_ = 0.541, *p* = 0.708, $\eta_{p}^{2}$ = 0.111) nor the less affected lower limb (F_4;26.903_ = 1.172, *p* = 0.345, $\eta_{p}^{2}$ = 0.148).

The measures derived from the more and the less affected lower limbs were separately analyzed, since the following measures were, in the tSCS-off condition, significantly lower in the more affected lower limb: (i) the median LEMS (more affected, 18.0 (interquartile range (IQR): 15.5–20); less affected, 21.5 (19.75–25.0), z = −2.673, *p* = 0.008, r = 0.845), (ii) the ankle ROM during active rhythmic movements (F1; 38.548 = 22.978, *p* < 0.001, $\eta_{p}^{2}$ = 0.373), and (iii) the EMG activities of TA during dorsiflexion (F1;37.076 = 7.846, *p* = 0.008, $\eta_{p}^{2}$ = 0.175), as well as of MG during plantar flexion (F1;37.181 = 10.043, *p* = 0.003, $\eta_{p}^{2}$ = 0.213).

All group results are detailed in Table S1.

### 3.2. Spinal Reflex Activity

**Figure 2:**
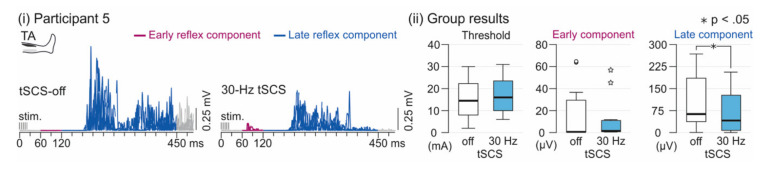
**Effect of tSCS on spinal reflex activity.** (**i**) Rectified electromyographic (EMG) responses of tibialis anterior (TA) to distal tibial nerve stimulation, applied to evoke a spinal reflex in the tSCS-off and the 30-Hz tSCS conditions in one participant. Early reflex components (purple lines) were identified within time windows of 60–120 ms post-stimulation train onset (stim.), and late reflex components (blue lines) within 120–450 ms. Shown are four (tSCS-off; one reflex excluded due to pre-activation) or five repetitions superimposed; participant 5, less affected lower limb. Note the shorter duration of the late reflex component under tonic 30-Hz tSCS. (**ii**) Group results of spinal reflex thresholds and TA EMG-root mean square (RMS) values associated with the early and late reflex component, respectively, during tSCS-off and 30-Hz tSCS conditions. The EMG-RMS of the late component was significantly reduced under tonic 30-Hz tSCS (asterisk; *p* < 0.05). Box plots represent medians by bold horizontal lines within boxes that span the interquartile range (IQR). Whiskers extend to the smallest and largest values that are not outliers (here, values between 1.5 and 3 times the IQR of the upper quartile; shown as separately plotted points) or extreme values (>3 times the IQR of the upper quartile; white asterisks).

Tonic 30-Hz tSCS did not alter the spinal reflex threshold (F_1;21.142_ = 0.493, *p* = 0.490, $\eta_{p}^{2}$ = 0.023) nor the EMG-RMS of the early reflex component (F_1;22.668_ = 0.775, p = 0.388, $\eta_{p}^{2}$ = 0.033; Figure 2, Table S2). On the other hand, it significantly reduced the EMG-RMS of the late reflex component (F_1;21.952_ = 6.337, *p* = 0.020, $\eta_{p}^{2}$ = 0.224). Lower limb (levels: more and less affected) was not a significant factor for any of the three measures (threshold, F_1;21.500_ = 0.262, *p* = 0.614, $\eta_{p}^{2}$ = 0.012; early component, F_1;23.935_ < 0.001, *p* = 0.988, $\eta_{p}^{2}$ < 0.001; late component, F_1;22.330_ = 0.037, *p* = 0.848, $\eta_{p}^{2}$ = 0.002). Further, no interaction between tSCS condition (tSCS-off, 30-Hz tSCS) and lower limb (levels: more and less affected) existed (threshold, F_1;21.142_ = 0.251, *p* = 0.621, $\eta_{p}^{2}$ = 0.012; early component, F_1;22.668_ = 0.009, *p* = 0.926, $\eta_{p}^{2}$ < 0.001; late component, F_1;21.952_ = 1.272, *p* = 0.272, $\eta_{p}^{2}$ = 0.002). All data are reported in Table S2.

### 3.3. Walking Performance

Walking performance, assessed using the FLOAT-BWS system and assistive devices as needed, showed considerable inter-individual differences, both in the baseline recordings without tSCS, as well as with respect to the effects of tonic tSCS at the three stimulation frequencies tested (individual values, see Table S3).

**Figure 3:**
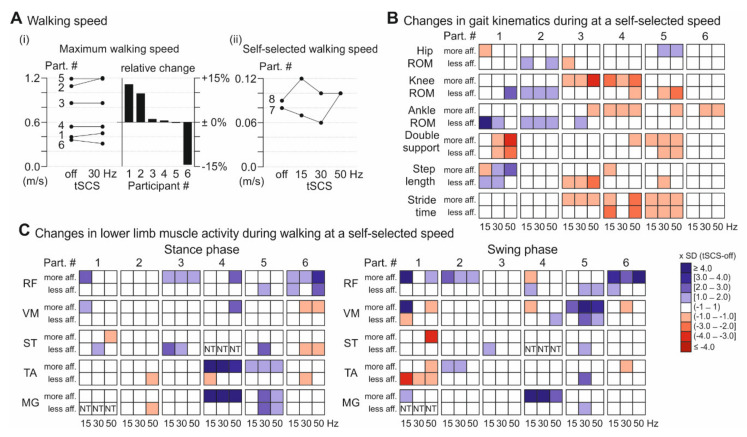
**Effect of tSCS on over-ground walking performance**. (**A**) Walking speed assessed on a 7 m track using the FLOAT-BWS system. (**i**) Absolute maximum walking speed of the six participants (see Section 2.2 Study protocol) completing the assessment during tSCS-off and 30-Hz tSCS, along with relative changes. (**ii**) Self-selected walking speed across tSCS conditions as indicated of the two participants (see Section 2.2 Study protocol) unable to maintain a constant speed over 7 m (both did not perform maximum walking speed assessment). (**B**) Changes in gait kinematics during 15-Hz, 30-Hz, and 50-Hz tSCS, given as multiples of the SD of the respective values in the tSCS-off condition (z-scores), and illustrated by the opacity of blue (indicating an increase) and red (decrease) boxes. Shown are data derived from the more and less affected lower limbs of participants 1–6 who walked at a constant self-selected speed across tSCS conditions. (**C**) Changes in lower limb muscle activation (RMS values) during stance (left) and swing phases (right). MG values of the less affected lower limb of participant 1, and ST values of the less affected lower limb of participant 4 are missing data. BWS, body-weight support; FLOAT, free levitation for over-ground active training; MG, medial gastrocnemius; RF, rectus femoris; ST, semitendinosus; TA, tibialis anterior; VM, vastus medialis.

Maximum walking speed without tSCS, as well as under tonic 30-Hz tSCS, was assessed in participants 1–6 (Figure 3A(i); not tested in participants 7 and 8, see Methods) and no significant group-effect between conditions was found (z = 0.734, *p* = 0.463, r = 0.212). Individual maximum walking speed was increased by 12.7% (+0.05 m/s) and 9.6% (+0.11 m/s) in participants 1 and 2, respectively, and reduced during stimulation by 14.4% (−0.05 m/s) in participant 6. Participants 3–5 showed no differences during tSCS-on compared to tSCS-off (+0.9%, +0.01 m/s; +0.4%, 0.00 m/s; −0.2%, 0.00 m/s). In the assessment of gait kinematics and lower limb muscle EMG activity, the six participants were able to maintain a constant (±10%) self-selected walking speed across tSCS conditions (tSCS-off, 15-Hz, 30-Hz, and 50-Hz tSCS; Table S3), while participants 7 and 8 completed the 7 m at variable, individually possible speed in the different conditions. The self-selected speed was increased during tonic tSCS at all three stimulation frequencies in participant 8 and reduced during 15-Hz and 30-Hz tSCS, and increased during 50-Hz tSCS, in participant 7 (Figure 3A(ii)).

For participants 1–6, with constant self-selected walking speed, there was no group effect of tSCS on kinematic and EMG outcome measures (for detailed statistics see Table S3), except for the stride time in the more affected leg, which was shorter during tonic stimulation (tSCS-off, median (IQR): 1.84 (1.36–3.22); 15 Hz: 1.75 (1.28–2.99); 30Hz: 1.78 (1.28–3.10); 50 Hz: 1.84 (1.27–2.85); χ^2^(3) = 9.8, *p* = 0.041). An overview of kinematics and muscle activity is provided for participants 1–6 in Figure 3B and 3C, and this revealed individual changes during tSCS, whereof some are briefly highlighted in the following. Participants 1 and 2 generally showed increased ROMs in one or more lower limb joints under tonic stimulation, and participant 1 additionally demonstrated decreased double limb support and increased step length. Participants 3–5 exhibited decreases in ROM, step length, and stride time during tSCS. Double limb support was additionally reduced in participant 5. Muscle activity of RF was enhanced during tSCS in the swing phase in participant 2 and in the stance phase in participant 3. Participants 1, 5, and 6 also exhibited augmented activity in RF or the synergistic VM, however, closer inspection revealed that the increase was attributable to stimulus triggered responses superimposed on the naturally generated EMG activities. TA and MG activity was increased under tSCS during stance in participant 5, whereas participant 1 exhibited decreased TA activity during swing. Participant 4 showed clonus-like activity in the ankle joint of the more affected limb during stimulation, resulting in increased muscle activity of the TA and MG.

In participants 7 and 8, with fluctuations of self-selected speed, mean hip and knee ROMs, as well as phases of double limb support were reduced in both lower limbs under tonic tSCS across the stimulation frequencies (Table S3), and in participant 8, the mean step length was additionally augmented. Both participants walked the 7 m with less variation in double limb support, stride time, and step length across tSCS frequencies, except for the step length of the more affected lower limb of participant 7 at 15 Hz, and of his less affected lower limb at 30 Hz (Table S3).

### 3.4. Subjective Reports

tSCS at 15 Hz, 30 Hz, and 50 Hz with targeted stimulation amplitudes was generally well tolerated by all participants with the exception of participant 7 in the first study session (stimulation amplitude hence set to 0.65 times the PRM-reflex threshold, a level clear below the threshold of discomfort). In the second study session, participant 7 reported no discomfort when being stimulated with the target amplitude. Across participants, no differences existed between comfort as well as performance perception between ankle control tests without and with tSCS (median VAS score (IQR), comfort: tSCS-off, 68 (55.8–84.5); tSCS-on, 73.5 (54.3–89.3); z = 0.059, *p* = 0.953, r = 0.019; performance: tSCS-off, (70 (51.5–78.3); tonic tSCS, 77 (57.0–85.0); z = 1.487, *p* = 0.137, r = 0.470). During walking, comfort perception did not differ between tSCS-off and tSCS-on conditions (62 (48.0–72.0) and 63 (53.0–82.5); z = 1.461, *p* = 0.144, r = 0.653). However, the performance perception during stimulation was significantly increased under tonic tSCS (65 (47.0–71.0) and 71 (61.0–79.5), z = 2.023, *p* = 0.043, r = 0.905).

## 4. Discussion

Single sessions of tonic tSCS produced modulatory effects on lower limb motor activity within minutes of application on group-level in individuals with chronic, sensorimotor iSCI. During rhythmic voluntary ankle movements, maximum dorsiflexion was significantly enhanced in the more affected lower limb during 30-Hz stimulation compared to tSCS-off. Despite a concomitant reduction of maximum plantar flexion, this resulted in an augmented active ROM. No effects on rhythmic ankle movements were observed for 15-Hz and 50-Hz tSCS. Furthermore, coordination of ankle joint movements was unchanged during stimulation across the frequencies. Electrophysiological recordings of a polysynaptic spinal reflex demonstrated, for the first time, an immediate interaction of tSCS and the spinal interneuronal networks associated with locomotion. There was no group effect of tSCS on locomotor function in a subset of eight participants, with high levels of inter-individual variability. Among the eight participants, the three with the lowest (score 13) and one with the maximum WISCI II scores showed increased maximum walking speed (participants 1 and 2) or more continuous (participants 7 and 8) and faster stepping (participant 8) at a self-selected speed. Some participants exhibited reduced ROM, step lengths, and strides times during tSCS.

The target neural structures of tSCS, applied over the lumbar spinal cord, are the large-to-medium diameter afferent fibers in posterior roots originating in distant dermatomes and myotomes, and are comparable to target structures of eSCS [17,19,20,28,29,69]. Here, stimulation of these target structures was verified by the elicitation of PRM reflexes with single stimulation pulses and their suppression and gradual recovery when using a paired-pulse paradigm [36,70]. Stimulation at 15 Hz, 30 Hz, and 50 Hz was applied, with an amplitude below the threshold for the elicitation of PRM reflexes at rest, thus not reflexively depolarizing alpha-motoneurons, while providing repetitive, multisegmental afferent input to the spinal cord [16,36]. The stimulation of afferent structures within the L2–S2 posterior roots during tonic tSCS was further supported by the occurrence of tingling sensations in the respective lower extremity dermatomes reported by some of the participants [34]. Repetitive afferent input to spinal networks generated by SCS is hypothesized to modulate spinal excitability, regulate activity in segmental spinal circuitries [34,40], and enhance their responsiveness to remaining descending input, ultimately resulting in improved motor control after SCI [16,38,39].

In this study, we demonstrated that tSCS at 30 Hz improved voluntary control of the ankle joint in the more affected lower limb of participants with chronic iSCI. Active ankle ROM and voluntary dorsiflexion were immediately increased, by 2.9° ± 0.9° and 4.6° ± 0.9°, respectively. A previous study investigated the effects of one-month Lokomat training on voluntary ankle movements in an iSCI cohort comparable to this study (11.80 ± 2.54 years post-injury, WISCI II 9–20) [71]. Post-training, dorsiflexion movements were improved, reflected by an increase of the active ROM by 5.1° ± 1.6, which is in a similar range as the tSCS-induced improvements in this study. Voluntary ankle control has been shown to be substantially improved with eSCS at 25–30 Hz, which enabled ankle movements in paralyzed muscles after motor complete SCI, including relatively fine controlled dorsiflexion [16]. The lack of facilitation in the less affected lower limb in the participants of the present study was likely related to the significantly higher LEMS on this side, with three participants scoring the maximum of 25, and another five participants having scores ≥20. Additionally, in the baseline recording without tSCS, ankle ROM and EMG activity of TA during dorsiflexion were greater than on the more affected side. Together, this leads to the assumption that the participants had maintained a critical level of control over voluntary ankle movements in their less affected lower limbs, holding less potential for further improvement. Absolute plantar flexion angles were unchanged, or even decreased, for both legs across stimulation conditions in this study. The plantar flexion movement was in the direction of gravity, and EMG activities recorded from MG were at a relatively low level across tSCS-off and tSCS-on conditions, suggesting that the participants did not perform this movement phase at full voluntary capacity. This may also explain the lack of the hypothesized facilitation of the plantar flexion phase by 15-Hz tSCS. A decrease of maximal plantarflexion angle from the off-condition before, to the off-condition after, the intervention might additionally indicate that participants were less engaged in plantarflexion over time. Since there was no change over time for the other variables, fatigue can be excluded as an origin of this reduction. No effects on rhythmic ankle movements were also observed during 50-Hz tSCS, where previous studies demonstrated an antispastic effect [32,33,34]. While we also observed signs of an antispastic effect (i.e., reduced clonus-like activity when stimulation was applied at 50 Hz in participant 7 (cf. Figure 1A)), stimulation at 50 Hz did not translate into functional improvements of ankle motor control over the whole group in the first application. However, as clonus-like muscle activity was not present in the majority of patients during initial tSCS-off, spasticity might not have been a major problem for ankle control in the assessed cohort. Coordination of the ankle joint was unchanged during tSCS across all frequencies. However, previous studies employing similar tasks demonstrated that ankle dexterity is largely retained after iSCI, while the strength of muscles acting on the ankle joint is constrained resulting in a reduced ROM [49,72,73].

This study showed for the first time a modulatory effect of electrical SCS on spinal locomotor networks by means of electrophysiological measures of spinal reflex activity. The activity of this reflex is substantially altered after SCI, and was suggested to reflect the functional state of spinal locomotor circuits [41,42,45]. While the physiological early reflex component is generally reduced after severe SCI, an additional late component gradually occurs after SCI, and dominance of this late component is associated with higher degrees of walking impairment [41,42,45]. However, if appropriate proprioceptive input is provided in individuals with chronic motor complete SCI (e.g., in a physiological movement task) this effect can be temporarily reversed in a single session, and the late reflex component can be decreased, while the early component reappears [74]. Furthermore, locomotor training after SCI was shown to increase the early, and decrease the late, reflex component during walking [75] or at rest [42]. In this study, the late reflex component was decreased under tonic 30-Hz stimulation for the more and less affected leg, suggesting an interaction of the afferent input provided by tSCS with spinal locomotor networks, and a resulting modulation of their excitability. No significant group effect on the early reflex component existed, yet, in some of the participants, it was increased or reoccurred (the latter in participant 5 only) when the stimulation was applied (cf. Figure 2A). This probably reflects inter-individual differences in initial spinal excitability levels, and may indicate that the afferent input provided by tSCS did not modulate the spinal excitability level in a similar manner to movement-related afferent feedback during stepping. Another mechanism involved in the observed effect could potentially be the spasticity reducing impact of tSCS. A previous study reported that the late component in the TA EMG activity after cutaneous reflex elicitation was reduced during active plantarflexion in controls, and that this modulation was absent after iSCI, however, only if spasticity was present [76]. This may indicate that the inhibitory effects of tSCS, involved in the known antispastic impact of the stimulation (possibly mechanisms of presynaptic inhibition, post activation depression, reciprocal inhibition [26,32,33,34]), contributed to the suppression of the late reflex component. Since prolonged reflex activity was observed in individuals with spasticity [76], observations of shortened late reflex components further indicate that such mechanisms were involved in the reflex modulation (cf. Figure 2A). An antispastic effect for tSCS and eSCS has been reported for stimulation frequencies around 50 Hz [26,32,33,34], but also for lower frequencies in eSCS [77]. As both tSCS, and the electrical stimulation to elicit the spinal reflex, share common stimulated neuronal structures [34,41], suppression of the spinal reflex caused by antidromic volleys following the posterior root stimulation may have led to decreases in amplitudes of the reflex components. Yet, likely only to a minor extent, considering the suppression of the late reflex component only across all participants, as well as the facilitation or recurrence of the early component in some participants.

There was no group effect of tSCS on walking ability, and high variability between participants was observed. The only statistical significance across participants 1–6 involved a reduction of the stride time in the more affected leg, which does probably not represent a clinically relevant change, as the highest difference between two conditions was 0.09 s. Whereas some participants showed enhanced maximum walking speed, increased joint ROMs, as well as decreased double limb support during tSCS, and can hence be considered as treatment responders, others showed only minor changes, or even reduced ROMs. Specifically, the increase in ankle ROM under tonic stimulation when participants were in the supine position did not generally translate to a similar increase during walking; nevertheless, this was present in responder participants 1 and 2 who increased their maximum walking speed during 30-Hz tSCS by, or beyond, the minimally clinically important difference of 0.05 m/s for individuals with SCI [78]. Participants 7 and 8, who walked with high kinematic variability and were unable to maintain a constant walking speed across repetitions, exhibited more continuous stepping when tSCS was applied. Notably, participants 1, 7, and 8 had WISCI II scores of 13, i.e., the lowest scores among the eight participants completing the walk tests, indicating that individuals with higher walking impairments are more likely treatment responders. Several other factors may have contributed to the variable group results. Walking is a much more complex motor task than rhythmic single joint movements, locomotor deficits may be highly variable between participants [79], and possible deficit-specific improvements dilute group effects. Additionally, the stimulation may not hold the potential to target each deficit in an equally efficient manner. Furthermore, all participants were in the chronic stage of recovery, and likely had difficulties to acutely adopt their walking strategies during a single session, and within the relatively short track of 7 m. Four of the eight participants had maximum WISCI II scores of 20, with probably even less space for further immediate improvement. It needs to be determined if individually optimized stimulation parameters and multiple applications can enhance the effects of tSCS on ambulation, and increase the number of treatment responders [36,80], as shown for eSCS [12,13,14]. The higher subjective performance ratings during walking under tonic stimulation may have been related to the co-activation of the paraspinal and abdominal muscles, leading to a perceived increased stabilization of the trunk. Yet, in some participants, this co-activation may have resulted in a more rigid gait, perhaps partially explaining the findings of reduced ROM, and highlighting the need of familiarization to the stimulation. Most participants were additionally unexperienced with the BWS setup, and familiarization to the system was possibly not completed in the first session.

In participants 9 and 10, PRM reflexes could not be elicited in the BWS standing position, with additional assistance of a walker required to stabilize equilibrium. Both participants exhibited a slightly forward bent posture during standing. It was previously shown that PRM reflex thresholds are generally higher in the standing than the supine position, and that changes in the volume conductor in between the paraspinal and abdominal electrodes, as in alterations of posture (e.g., a forward bend), can substantially influence the effects of tSCS [81].

Despite applying stimulation amplitudes below the PRM reflexes threshold in the standing position, stimulus-triggered responses were superimposed on the EMG signals of the thigh muscles of participants 1, 5, and 6 during walking. Additional afferent input and posture alterations during the dynamic task possibly changed response thresholds, as well as activation sites of tSCS to the intervertebral foramina, where posterior and anterior roots approach each other to form the spinal nerve [81,82]. The clonus-like activity in TA and MG of participant 4 during walking under tSCS (not observed during ankle assessments) was probably caused by the combination of afferent input from tSCS and the proprioceptive feedback related to the motor task.

A limitation of the current study, and a general challenge for clinical studies using tSCS, is the lack of a sham intervention with confirmed ineffectiveness [33,34,83]. Yet, in the assessment of rhythmic ankle movements, effects were only observed in the more affected lower limb with significantly lower LEMS, indicating that placebo effects likely played a minor role. The findings of the study are further limited due to the low sample size, especially in the walking condition. A limitation of tSCS itself is the constrained target cohort among individuals with SCI, given by the exclusion criteria comprising osteosynthesis material at the site of stimulation, as well as caudal injury sites that are regularly accompanied by secondary peripheral lesions. The order of the assessments (ankle control tasks, spinal reflex, walking tests) might have influenced the results, because participants gained experience with tSCS. However, this was probably only present to a minor extent, since the biggest effects were observed in the first assessment.

## 5. Conclusions

This study demonstrated that tonic tSCS can acutely facilitate residual voluntary ankle control after chronic iSCI, and modulates spinal locomotor networks, i.e., polysynaptic spinal reflex behavior. Effects on locomotion were variable across participants, yet, the ones with lower ambulatory function showed increased maximum walking speed or more continuous stepping in the presence of tSCS. This indicates that effects on walking performance likely also depend on the degree of impairment of the baseline walking ability. Individually tailored stimulation parameter settings, as well as multiple applications of the intervention together with task-specific training paradigms, may lead to enhanced clinical outcomes.

## Figures and Tables

**Table 1:** Neurological status of the participants according to the International Standards for Neurological Classification of Spinal Cord Injury.

Nr.	Sex	Age (y)	Neurol. Level of SCI	Time Post-SCI (y)	LEMS Total (L/R)	PP Sensory Score Total (L/R)	LT Sensory Score Total (L/R)	WISCI II Score	FLOAT-BWS (%)
1	M	28	C5	8	5/25	32/32	32/32	13	7
2	M	53	C3	38	25/17	29/30	30/30	20	7
3	M	65	T10	15	16/20	47/49	45/45	20	7
4	M	40	C6	4	21/20	20/18	26/25	16	10
5	M	45	C7	15	14/15	29/26	28/27	20	6
6	M	48	C5	8	20/22	31/31	31/31	20	6
7	M	31	T4	13	19/19	40/36	40/40	13	45
8	M	40	C7	5	17/21	22/22	39/39	13	6
9	F	40	C4	4	23/21	31/31	31/31	9	NT
10	M	64	T3	6	25/23	39/37	44/45	13	NT

BWS, body weight support; FLOAT, free levitation for over-ground active training [48]; LEMS, lower extremity motor score (max. 25 per leg); LT, light touch (max. 56 per side); PP, pin prick (max. 56 per side); Neurol., neurological; Part., participant; WISCI, walking index for spinal cord injury [47]; y, years; SCI, spinal cord injury.

## Supplementary Materials

Supplementary materials can be found at https://www.mdpi.com/2077-0383/9/11/3541/s1. Table S1: Ankle control assessments, Table S2: Spinal reflex activity, Table S3: Walk tests.
