# Supplementary material for: Immediate Effects of Transcutaneous Spinal Cord Stimulation on Motor Function in Chronic, Sensorimotor Incomplete Spinal Cord Injury
Source: J Clin Med. 2020 Nov 2;9(11):3541. doi: 10.3390/jcm9113541 (PMC7694146; doi:10.3390/jcm9113541)
Supplement: Supplementary file 1 [file jcm-09-03541-s001.zip › Table S1 done.docx]

**Table S1.** Ankle control assessments.

| **(A) Rhythmic unilateral dorsiflexion-plantar flexion movements.** | | |
| --- | --- | --- |
| **Ankle range of motion (mean degree ± SE)** | | |
|  | **More affected lower limb** | **Less affected lower limb** |
| **tSCS condition x movement rate** | F_8;89.009_ = 0.121, p = .998, $\eta_{p}^{2}$ = 0.011 | F_8;123.002_ = 0.164, p = 0.995, $\eta_{p}^{2}$ = 0.011 |
| **tSCS condition** | F_4;89.016_ = 4.368, p = .003, $\eta_{p}^{2}$ = 0.164 | F_4;123.011_ = 2.522, p = 0.044, $\eta_{p}^{2}$ = 0.076 |
| tSCS-off | 21.6° ± 2.4° | 30.2 ± 2.7 |
| 15-Hz tSCS | 22.7° ± 2.2° | 28.6 ± 2.3 |
| 30-Hz tSCS | 24.7° ± 2.3° | 29.8 ± 2.6 |
| 50-Hz tSCS | 22.9° ± 2.3° | 30.3 ± 2.4 |
| tSCS-off_rep_ | 20.7° ± 2.5° | 28.3 ± 2.6 |
| ***Post-hoc pairwise comparisons (mean differences ± SE)*** | | |
| tSCS-off: 15-Hz tSCS | 1.2° ± 0.9°, p = 1.000 | 1.5 ± 1.0, p = 1.000 |
| tSCS-off: 30-Hz tSCS | 2.9° ± 0.9°, p = 0.013 | 0.4 ± 1.0, p = 1.000 |
| tSCS-off: 50-Hz tSCS | 1.4° ± 0.9°, p = 1.000 | 0.1 ± 1.0, p = 1.000 |
| tSCS-off: tSCS-off_rep_ | 0.5° ± 0.9°, p = 1.000 | 2.7 ± 1.1, p = 0.124 |
| 15-Hz tSCS: 30-Hz tSCS | 1.7° ± 0.9°, p = 0.537 | 1.2 ± 1.0, p = 1.000 |
| 15-Hz tSCS: 50-Hz tSCS | 0.2° ± 0.9°, p = 1.000 | 1.6 ± 1.0, p = 1.000 |
| 15-Hz tSCS: tSCS-off_rep_ | 1.7° ± 0.9°, p = 0.663 | 1.1 ± 1.1, p = 1.000 |
| 30-Hz tSCS: 50-Hz tSCS | 1.5° ± 0.9°, p = 0.854 | 0.5 ± 1.0, p = 1.000 |
| 30-Hz tSCS: tSCS-off_rep_ | 3.4° ± 0.9°, p = 0.003 | 2.3 ± 1.1, p = 0.307 |
| 50-Hz tSCS: tSCS-off_rep_ | 1.9° ± 0.9°, p = 0.395 | 2.8 ± 1.1, p = 0.096 |
| **Maximum dorsiflexion angle (mean degree ± SE)** | | |
|  | **More affected lower limb** | **Less affected lower limb** |
| **tSCS condition x movement rate** | F_8;89.007_ = 0.114, p = .999, $\eta_{p}^{2}$ = 0.010 | F_8;123.001_ = 0.148, p = 0.997, $\eta_{p}^{2}$ = 0.010 |
| **tSCS condition** | F_4;89.011_ = 6.779, p < .001, $\eta_{p}^{2}$ = 0.234 | F_4;123.005_ = 1.390, p = 0.241, $\eta_{p}^{2}$ = 0.043 |
| tSCS-off | -38.2 ± 3.4 | -28.7 ± 3.4 |
| 15-Hz tSCS | -36.8 ± 2.8 | -28.4 ± 3.0 |
| 30-Hz tSCS | -34.2 ± 2.9 | -27.5 ± 3.2 |
| 50-Hz tSCS | -36.7 ± 2.9 | -27.8 ± 3.0 |
| tSCS-off_rep_ | -38.5 ± 3.4 | -28.2 ± 3.5 |
| ***Post-hoc pairwise comparisons (mean differences ± SE)*** | | |
| tSCS-off: 15-Hz tSCS | 1.8° ± 0.9°, p = 0.493 | NA |
| tSCS-off: 30-Hz tSCS | 4.6° ± 0.9°, p < 0.001 | NA |
| tSCS-off: 50-Hz tSCS | 1.9° ± 0.9°, p = 0.409 | NA |
| tSCS-off: tSCS-off_rep_ | 0.8° ± 1.0°, p = 1.000 | NA |
| 15-Hz tSCS: 30-Hz tSCS | 2.7° ± 0.9°, p = 0.041 | NA |
| 15-Hz tSCS: 50-Hz tSCS | 0.1° ± 0.9°, p = 1.000 | NA |
| 15-Hz tSCS: tSCS-off_rep_ | 1.1° ± 1.0°, p = 1.000 | NA |
| 30-Hz tSCS: 50-Hz tSCS | 2.7° ± 0.9°, p = 0.052 | NA |
| 30-Hz tSCS: tSCS-off_rep_ | 3.8° ± 1.0°, p = 0.002 | NA |
| 50-Hz tSCS: tSCS-off_rep_ | 1.1° ± 1.0°, p = 1.000 | NA |
| **Maximum plantar flexion angle (mean degree ± SE)** | | |
|  | **More affected lower limb** | **Less affected lower limb** |
| **tSCS condition x movement rate** | F_8;89.00_ = 0.745, p = 0.651, $\eta_{p}^{2}$ = 0.063 | F_8;123.001_ = 0.289, p = 0.968, $\eta_{p}^{2}$ = 0.018 |
| **tSCS condition** | F_4;89.005_ = 3.557, p = 0.010, $\eta_{p}^{2}$ = 0.138 | F_4;123.003_ = 8.125, p < 0.001, $\eta_{p}^{2}$ = 0.209 |
| tSCS-off | -59.6 ± 1.8 | -58.9 ± 1.7 |
| 15-Hz tSCS | -59.6 ± 1.5 | -57.0 ± 1.6 |
| 30-Hz tSCS | -58.9 ± 1.5 | -57.3 ± 1.5 |
| 50-Hz tSCS | -59.6 ± 1.5 | -58.1 ± 1.7 |
| tSCS-off_rep_ | -59.2 ± 1.8 | -56.6 ± 1.7 |
| ***Post-hoc pairwise comparisons (mean differences ± SE)*** | | |
| tSCS-off: 15-Hz tSCS | 0.7° ± 0.5°, p = 1.000 | 1.9 ± 0.4, p < 0.001 |
| tSCS-off: 30-Hz tSCS | 1.7° ± 0.5°, p = 0.008 | 1.5 ± 0.4, p = 0.001 |
| tSCS-off: 50-Hz tSCS | 0.5° ± 0.5°, p = 1.000 | 0.8 ± 0.4, p = 0.439 |
| tSCS-off: tSCS-off_rep_ | 1.3° ± 0.5°, p = 0.147 | 1.8 ± 0.4, p < .001 |
| 15-Hz tSCS: 30-Hz tSCS | 1.1° ± 0.5°, p = 0.363 | 0.3 ± 0.4, p = 1.000 |
| 15-Hz tSCS: 50-Hz tSCS | 0.1° ± 0.5°, p = 1.000 | 1.1 ± 0.4, p = 0.065 |
| 15-Hz tSCS: tSCS-off_rep_ | 0.6° ± 0.5°, p = 1.000 | 0.1 ± 0.4, p = 1.000 |
| 30-Hz tSCS: 50-Hz tSCS | 1.2° ± 0.5°, p = 0.210 | 0.7 ± 0.4, p = 0.605 |
| 30-Hz tSCS: tSCS-off_rep_ | 0.4° ± 0.5°, p = 1.000 | 0.3 ± 0.4, p = 1.000 |
| 50-Hz tSCS: tSCS-off_rep_ | 0.7° ± 0.5°, p = 1.000 | 1.0 ± 0.4, p = 0.151 |
| **Tibialis anterior activity during dorsiflexion (mean RMS ± SE, µV)** | | |
|  | **More affected lower limb** | **Less affected lower limb** |
| **tSCS condition x movement rate** | F_8;88.024_ = 0.125, p = 0.998, $\eta_{p}^{2}$ = 0.011 | F_8;123.001_ = 0.345, p = 0.947, $\eta_{p}^{2}$ = 0.022 |
| **tSCS condition** | F_4;88.042_ = 2.004, p = 0.101, $\eta_{p}^{2}$ = 0.083 | F_4;123.005_ = 2.673, p = 0.035, $\eta_{p}^{2}$ = 0.080 |
| tSCS-off | 58.8 ± 8.6 | 96.3 ± 14.3 |
| 15-Hz tSCS | 54.0 ± 5.0 | 92.2 ± 12.8 |
| 30-Hz tSCS | 60.3 ± 6.5 | 98.1 ± 14.4 |
| 50-Hz tSCS | 55.9 ± 6.6 | 99.3 ± 13.9 |
| tSCS-off_rep_ | 50.2 ± 6.4 | 91.3 ± 14.3 |
| ***Post-hoc pairwise comparisons (mean differences ± SE, µV)*** | | |
| tSCS-off: 15-Hz tSCS | NA | 4.1 ± 4.0, p = 1.000 |
| tSCS-off: 30-Hz tSCS | NA | 1.8 ± 4.0, p = 1.000 |
| tSCS-off: 50-Hz tSCS | NA | 3.0 ± 4.0, p = 1.000 |
| tSCS-off: tSCS-off_rep_ | NA | 8.6 ± 4.1, p = .383 |
| 15-Hz tSCS: 30-Hz tSCS | NA | 5.9 ± 4.0, p = 1.000 |
| 15-Hz tSCS: 50-Hz tSCS | NA | 7.1 ± 4.0, p = 0.766 |
| 15-Hz tSCS: tSCS-off_rep_ | NA | 4.5 ± 4.1, p = 1.000 |
| 30-Hz tSCS: 50-Hz tSCS | NA | 1.2 ± 4.0, p = 1.000 |
| 30-Hz tSCS: tSCS-off_rep_ | NA | 10.4 ± 4.1, p = 0.125 |
| 50-Hz tSCS: tSCS-off_rep_ | NA | 11.5 ± 4.1, p = 0.056 |
| **Medial gastrocnemius activity during plantarflexion (mean RMS ± SE, µV)** | | |
|  | **More affected lower limb** | **Less affected lower limb** |
| **tSCS condition x movement rate** | F_8;87.943_ = 0.246, p = .981, $\eta_{p}^{2}$ = 0.022 | F_8;122.997_ = 0.518, p = 0.841, $\eta_{p}^{2}$ = 0.033 |
| **tSCS condition** | F_4;88.037_ = 3.290, p = .015, $\eta_{p}^{2}$ = 0.130 | F_4;123.017_ = 8.127, p < 0.001, $\eta_{p}^{2}$ = 0.209 |
| tSCS-off | 27.1 ± 1.4 | 43.4 ± 4.2 |
| 15-Hz tSCS | 23.6 ± 1.7 | 34.9 ± 4.2 |
| 30-Hz tSCS | 23.6 ± 1.8 | 36.9 ± 4.1 |
| 50-Hz tSCS | 24.7 ± 1.5 | 40.2 ± 3.9 |
| tSCS-off_rep_ | 22.9 ± 1.0 | 33.0 ± 4.1 |
| ***Post-hoc pairwise comparisons (mean differences ± SE, µV)*** | | |
| tSCS-off: 15-Hz tSCS | 3.4 ± 1.6, p = 0.301 | 8.5 ± 2.4, p = 0.005 |
| tSCS-off: 30-Hz tSCS | 3.2 ± 1.6, p = 0.426 | 6.4 ± 2.4, p = 0.078 |
| tSCS-off: 50-Hz tSCS | 2.3 ± 1.6, p = 1.000 | 3.2 ± 2.4, p = 1.000 |
| tSCS-off: tSCS-off_rep_ | 5.7 ± 1.6, p = 0.007 | 12.9 ± 2.5, p < 0.001 |
| 15-Hz tSCS: 30-Hz tSCS | 0.2 ± 1.6, p = 1.000 | 2.0 ± 2.4, p = 1.000 |
| 15-Hz tSCS: 50-Hz tSCS | 1.1 ± 1.5, p = 1.000 | 5.3 ± 2.4, p = 0.276 |
| 15-Hz tSCS: tSCS-off_rep_ | 2.3 ± 1.6, p = 1.000 | 4.4 ± 2.5, p = 0.738 |
| 30-Hz tSCS: 50-Hz tSCS | 0.9 ± 1.6, p = 1.000 | 3.3 ± 2.4, p = 1.000 |
| 30-Hz tSCS: tSCS-off_rep_ | 2.5 ± 1.6, p = 1.000 | 6.5 ± 2.5, p = 0.098 |
| 50-Hz tSCS: tSCS-off_rep_ | 3.4 ± 1.6, p = 0.348 | 9.8 ± 2.5, p = 0.001 |
| **Deviation from target movement rate (%)** | | |
|  | **More affected lower limb** | **Less affected lower limb** |
| **tSCS condition x movement rate** | F_8;88.944_ = 0.834, p = 0.575, $\eta_{p}^{2}$ = 0.070 | F_8;122.945_ = 0.404, p = 0.916, $\eta_{p}^{2}$ = 0.026 |
| **tSCS condition** | F_4;89.028_ = 2.872, p = 0.027, $\eta_{p}^{2}$ = 0.114 | F_4;123.083_ = 0.820, p = 0.514, $\eta_{p}^{2}$ = 0.026 |
| tSCS-off | 11.2 ± 1.4 | 8.3 ± 1.6 |
| 15-Hz tSCS | 10.0 ± 1.6 | 6.9 ± 1.9 |
| 30-Hz tSCS | 14.6 ± 2.5 | 7.0 ± 1.1 |
| 50-Hz tSCS | 12.8 ± 2.1 | 7.4 ± 1.3 |
| tSCS-off_rep_ | 15.0 ± 3.0 | 8.7 ± 1.5 |
| **(B) Maximum ankle range of movement and unilateral ankle movements following an irregular sinusoidal trajectory** | | |
| **Normalized RMS-error (%)** | | |
|  | **More affected lower limb** | **Less affected lower limb** |
| **tSCS condition** | F_4;17.259_ = 0.541, p = 0.708, $\eta_{p}^{2}$ = 0.111 | F_4;26.903_ = 1.172, p = 0.345, $\eta_{p}^{2}$ = 0.148 |
| tSCS-off | 5.0 ± 0.8 | 4.6 ± 0.6 |
| 15-Hz tSCS | 5.5 ± 0.5 | 4.2 ± 0.5 |
| 30-Hz tSCS | 5.6 ± 0.6 | 4.3 ± 0.4 |
| 50-Hz tSCS | 5.2 ± 0.7 | 4.1 ± 0.4 |
| tSCS-off_rep_ | 4.9 ± 0.5 | 3.9 ± 0.3 |

RMS, root-mean-square.
