# Supplementary material for: Immediate Effects of Transcutaneous Spinal Cord Stimulation on Motor Function in Chronic, Sensorimotor Incomplete Spinal Cord Injury
Source: J Clin Med. 2020 Nov 2;9(11):3541. doi: 10.3390/jcm9113541 (PMC7694146; doi:10.3390/jcm9113541)
Supplement: Supplementary file 1 [file jcm-09-03541-s001.zip › Table S2 done.docx]

**Table S2.** Spinal reflex activity.

| **Threshold (mA; median and IQR)** |  |  |
| --- | --- | --- |
| **tSCS condition x lower limb (more/less affected)** | F_1;21.142_ = 0.251, p = 0.621, $\eta_{p}^{2}$ = 0.012 | |
| **lower limb (more/less affected)** | F_1;21.500_ = 0.262, p = 0.614, $\eta_{p}^{2}$ = 0.012 | |
| **tSCS condition** | F_1;21.142_ = 0.493, p = 0.490, $\eta_{p}^{2}$ = 0.023 | |
| tSCS-off | 14.5 (8.0–22.39) | |
| 30-Hz tSCS | 16.0 (10.0–23.5) | |
| **EMG-RMS of early reflex component (µV; median and IQR)** | | |
| **tSCS condition x lower limb (more/less affected)** | F_1;22.668_ = 0.009, p = 0.926, $\eta_{p}^{2}$ < 0.001 | |
| **lower limb (more/less affected)** | F_1;23.935_ < 0.001, p = 0.988, $\eta_{p}^{2}$ < 0.001 | |
| **tSCS condition** | F_1;22.668_ = 0.775, p = 0.388, $\eta_{p}^{2}$ = 0.033 | |
| tSCS-off | 0.7 (0.1–29.4) | |
| 30-Hz tSCS | 1.6 (0.4–11.0) | |
| **EMG-RMS of late reflex component (µV; median and IQR)** | | |
| **tSCS condition x lower limb (more/less affected)** | F_1;21.952_ = 1.272, p = 0.272, $\eta_{p}^{2}$ = 0.002 | |
| **lower limb (more/less affected)** | F_1;22.330_ = 0.037, p = 0.848, $\eta_{p}^{2}$ = 0.002 | |
| **tSCS condition** | F_1;21.952_ = 6.337, p = 0.020, $\eta_{p}^{2}$ = 0.224 | |
| tSCS-off | 62.9 (37.3–185.2) | |
| 30-Hz tSCS | 41.2 (7.9–127.0) | |

EMG, electromyographic; RMS, root-mean-square.
