# Supplementary material for: Immediate Effects of Transcutaneous Spinal Cord Stimulation on Motor Function in Chronic, Sensorimotor Incomplete Spinal Cord Injury
Source: J Clin Med. 2020 Nov 2;9(11):3541. doi: 10.3390/jcm9113541 (PMC7694146; doi:10.3390/jcm9113541)
Supplement: Supplementary file 1 [file jcm-09-03541-s001.zip › Table S3 done.docx]

**Table S3.** Walk tests.

| **(A) Walking speed and walking kinematics** | | | | | |
| --- | --- | --- | --- | --- | --- |
| **Maximum speed (m/s)** | | | | | |
|  |  | **tSCS-Off** | **15 Hz** | **30 Hz** | **50 Hz** |
| Participant 1 |  | 0.40 | NT | 0.45 | NT |
| Participant 2 |  | 1.09 | NT | 1.20 | NT |
| Participant 3 |  | 0.86 | NT | 0.86 | NT |
| Participant 4 |  | 0.53 | NT | 0.54 | NT |
| Participant 5 |  | 1.19 | NT | 1.19 | NT |
| Participant 6 |  | 0.36 | NT | 0.31 | NT |
| Participant 7 |  | NT | NT | NT | NT |
| Participant 8 |  | NT | NT | NT | NT |
| **Self-selected speed (m/s)** | | | | | |
|  |  | **tSCS-Off** | **15 Hz** | **30 Hz** | **50 Hz** |
| Participant 1 |  | 0.18 | 0.19 | 0.19 | 0.19 |
| Participant 2 |  | 0.90 | 0.93 | 0.93 | 0.94 |
| Participant 3 |  | 0.77 | 0.78 | 0.78 | 0.77 |
| Participant 4 |  | 0.42 | 0.42 | 0.41 | 0.45 |
| Participant 5 |  | 1.07 | 1.13 | 1.16 | 1.10 |
| Participant 6 |  | 0.30 | 0.32 | 0.32 | 0.29 |
| Participant 7 |  | 0.08 | 0.07 | 0.06 | 0.10 |
| Participant 8 |  | 0.09 | 0.12 | 0.10 | 0.10 |
| **Hip range of motion (degree; mean ± SD)** | | | | | |
|  | **Lower limb** | **tSCS-off** | **15 Hz** | **30 Hz** | **50 Hz** |
| Participant 1 | more affected | 20.9 ± 0.9 | 19.7 ± 1.8 | 20.5 ± 1.7 | 20.5 ± 2.1 |
|  | less affected | 19.0 ± 1.9 | 19.8 ± 1.7 | 9.2 ± 1.3 | 19.4 ± 2.0 |
| Participant 2 | more affected | 23.8 ± 2.5 | 24.6 ± 3.3 | 24.5 ± 2.7 | 25.5 ± 2.3 |
|  | less affected | 31.3 ± 2.0 | 30.9 ± 1.9 | 34.1 ± 3.6 | 32.5 ± 2.6 |
| Participant 3 | more affected | 28.1 ± 2.3 | 28.6 ± 2.0 | 27.7 ± 2.4 | 28.9 ± 3.2 |
|  | less affected | 36.0 ± 2.6 | 32.7 ± 2.6 | 34.4 ± 2.5 | 33.4 ± 3.0 |
| Participant 4 | more affected | 11.4 ± 2.1 | 9.7 ± 1.1 | 11.6 ± 1.7 | 9.5 ± 1.7 |
|  | less affected | 13.4 ± 0.9 | 13.4 ± 2.1 | 12.9 ± 1.1 | 13.0 ± 2.3 |
| Participant 5 | more affected | 16.2 ± 1.5 | 15.9 ± 2.0 | 18.2 ± 1.3 | 18.2 ± 1.1 |
|  | less affected | 16.1 ± 2.5 | 16.5 ± 2.2 | 17.1 ± 2.4 | 14.7 ± 1.3 |
| Participant 6 | more affected | 9.2 ± 1.4 | 9.0 ± 2.9 | 8.8 ± 2.1 | 10.3 ± 1.9 |
|  | less affected | 6.7 ± 1.2 | 7.9 ± 2.0 | 7.9 ± 2.3 | 7.8 ± 1.7 |
| Friedman test | more affected | χ^2^(3) = 2.6, p = 0.916 | | | |
|  | less affected | χ^2^(3) = 1.4, p = 1.000 | | | |
| Participant 7 | more affected | 10.0 ± 0.9 | 6.4 ± 1.5 | 7.9 ± 1.3 | 6.8 ± 1.1 |
|  | less affected | 9.9 ± 1.2 | 6.8 ± 1.3 | 8.9 ± 1.3 | 9.2 ± 1.5 |
| Participant 8 | more affected | 23.0 ± 2.1 | 20.6 ± 2.1 | 18.8 ± 2.1 | 19.4 ± 2.8 |
|  | less affected | 31.3 ± 2.3 | 23.7 ± 2.5 | 23.5 ± 2.7 | 26.4 ± 1.8 |
| **Knee range of motion (degree; mean ± SD)** | | | | | |
|  | **Lower limb** | **tSCS-off** | **15 Hz** | **30 Hz** | **50 Hz** |
| Participant 1 | more affected | 50.3 ± 1.5 | 51.3 ± 1.9 | 50.0 ± 1.4 | 50.7 ± 1.4 |
|  | less affected | 39.4 ± 2.2 | 38.2 ± 3.2 | 41.3 ± 3.0 | 43.7 ± 2.3 |
| Participant 2 | more affected | 37.3 ± 3.5 | 36.6 ± 3.1 | 34.6 ± 3.2 | 34.7 ± 1.3 |
|  | less affected | 47.0 ± 3.5 | 51.1 ± 5.0 | 51.6 ± 3.1 | 53.5 ± 2.7 |
| Participant 3 | more affected | 59.9 ± 2.1 | 56.0 ± 4.2 | 56.9 ± 2.9 | 51.6 ± 3.6 |
|  | less affected | 59.4 ± 1.7 | 59.4 ± 1.8 | 59.7 ± 1.8 | 58.7 ± 1.5 |
| Participant 4 | more affected | 42.1 ± 2.3 | 36.6 ± 2.7 | 38.5 ± 4.1 | 36.5 ± 4.1 |
|  | less affected | 44.4 ± 6.9 | 38.9 ± 4.0 | 40.2 ± 3.3 | 36.0 ± 3.3 |
| Participant 5 | more affected | 38.1 ± 2.8 | 40.9 ± 1.9 | 36.4 ± 3.5 | 38.7 ± 2.7 |
|  | less affected | 46.5 ± 2.2 | 46.7 ± 1.3 | 42.4 ± 2.1 | 41.7 ± 1.8 |
| Participant 6 | more affected | 15.6 ± 3.2 | 15.4 ± 2.5 | 16.8 ± 1.6 | 17.4 ± 2.3 |
|  | less affected | 24.1 ± 4.0 | 27.3 ± 2.1 | 26.7 ± 4.3 | 26.3 ± 3.4 |
| Friedman test | more affected | χ^2^(3) = 2.0, p = 1.000 | | | |
|  | less affected | χ^2^(3) = 1.8, p = 1.000 | | | |
| Participant 7 | more affected | 13.7 ± 2.0 | 10.4 ± 3.5 | 10.0 ± 3.1 | 12.5 ± 2.9 |
|  | less affected | 25.5 ± 1.7 | 20.6 ± 3.7 | 26.6 ± 2.9 | 25.5 ± 1.3 |
| Participant 8 | more affected | 43.7 ± 2.0 | 30.4 ± 2.9 | 30.3 ± 2.3 | 31.9 ± 2.6 |
|  | less affected | 34.6 ± 2.9 | 28.6 ± 2.8 | 27.9 ± 2.1 | 30.1 ± 2.3 |
| **Ankle range of motion (degree; mean ± SD)** | | | | | |
|  | **Lower limb** | **tSCS-off** | **15 Hz** | **30 Hz** | **50 Hz** |
| Participant 1 | more affected | 15.6 ± 1.6 | 14.8 ± 1.6 | 16.0 ± 1.8 | 15.8 ± 1.3 |
|  | less affected | 14.5 ± 1.5 | 23.6 ± 3.9 | 16.1 ± 2.6 | 15.9 ± 2.8 |
| Participant 2 | more affected | 14.6 ± 1.7 | 13.5 ± 1.3 | 13.7 ± 1.7 | 13.7 ± 2.3 |
|  | less affected | 19.3 ± 2.8 | 23.1 ± 3.5 | 24.0 ± 1.7 | 23.4 ± 2.5 |
| Participant 3 | more affected | 27.7 ± 1.7 | 27.2 ± 2.2 | 27.4 ± 1.8 | 25.7 ± 2.5 |
|  | less affected | 18.1 ± 1.0 | 18.3 ± 1.5 | 19.3 ± 1.4 | 18.0 ± 1.2 |
| Participant 4 | more affected | 20.6 ± 1.7 | 17.5 ± 1.4 | 18.0 ± 2.4 | 18.4 ± 1.6 |
|  | less affected | 23.3 ± 5.4 | 22.1 ± 2.8 | 24.3 ± 2.4 | 22.5 ± 2.9 |
| Participant 5 | more affected | 19.9 ± 2.3 | 18.0 ± 1.6 | 17.8 ± 2.5 | 17.1 ± 2.4 |
|  | less affected | 17.7 ± 1.6 | 17.2 ± 2.0 | 16.3 ± 2.1 | 16.1 ± 0.9 |
| Participant 6 | more affected | 13.6 ± 1.4 | 14.0 ± 1.3 | 12.1 ± 1.4 | 12.2 ± 1.7 |
|  | less affected | 12.7 ± 2.7 | 13.5 ± 2.9 | 11.0 ± 2.5 | 11.7 ± 2.0 |
| Friedman test | more affected | χ^2^(3) = 5.4, p = 0.290 | | | |
|  | less affected | χ^2^(3) = 3.0, p = 0.784 | | | |
| Participant 7 | more affected | 9.4 ± 1.4 | 12.4 ± 2.7 | 9.5 ± 1.4 | 9.9 ± 1.4 |
|  | less affected | 6.2 ± 1.2 | 8.7 ± 1.8 | 7.4 ± 0.9 | 7.2 ± 0.8 |
| Participant 8 | more affected | 32.1 ± 2.5 | 25.0 ± 3.9 | 22.3 ± 2.9 | 19.0 ± 3.3 |
|  | less affected | 38.2 ± 1.1 | 32.9 ± 3.6 | 34.0 ± 2.2 | 38.2 ± 1.4 |
| **Double limb support (%; mean ± SD (coefficient of variation))** | | | | | |
|  | **Lower limb** | **tSCS-off** | **15 Hz** | **30 Hz** | **50 Hz** |
| Participant 1 | more affected | 68.9 ± 3.7 | 66.2 ± 4.3 | 64.0 ± 2.5 | 60.9 ± 3.2 |
|  | less affected | 68.9 ± 4.1 | 66.4 ± 3.4 | 64.3 ± 4.2 | 60.1 ± 3.4 |
| Participant 2 | more affected | 29.6 ± 3.3 | 28.0 ± 2.2 | 28.6 ± 1.7 | 28.1 ± 4.2 |
|  | less affected | 29.6 ± 3.0 | 29.0 ± 3.5 | 28.7 ± 2.7 | 28.3 ± 4.4 |
| Participant 3 | more affected | 35.4 ± 2.7 | 33.7 ± 2.5 | 33.2 ± 2.5 | 33.4 ± 2.7 |
|  | less affected | 35.6 ± 3.2 | 33.8 ± 2.5 | 33.4 ± 3.1 | 33.7 ± 2.7 |
| Participant 4 | more affected | 45.5 ± 3.1 | 44.4 ± 5.1 | 46.6 ± 2.5 | 40.9 ± 2.7 |
|  | less affected | 45.4 ± 4.0 | 43.4 ± 4.6 | 46.8 ± 2.7 | 41.5 ± 2.9 |
| Participant 5 | more affected | 27.1 ± 3.2 | 23.6 ± 1.0 | 21.0 ± 4.3 | 23.3 ± 2.2 |
|  | less affected | 25.9 ± 3.2 | 23.1 ±1.6 | 21.1 ± 3.0 | 22.1 ± 3.2 |
| Participant 6 | more affected | 43.7 ± 4.1 | 43.8 ± 3.0 | 41.7 ± 4.7 | 44.6 ± 3.2 |
|  | less affected | 43.9 ± 5.6 | 43.7 ± 2.8 | 41.7 ± 4.9 | 44.7 ± 2.6 |
| Friedman test | more affected | χ^2^(3) = 5.4, p = 0.290 | | | |
|  | less affected | χ^2^(3) = 8.2, p = 0.084 | | | |
| Participant 7 | more affected | 69.9 ± 8.6 (0.12) | 66.9 ± 5.6 (0.08) | 67.9 ± 5.9 (0.09) | 59.6 ± 3.8 (0.06) |
|  | less affected | 69.0 ± 8.2 (0.12) | 66.9 ± 7.5 (0.11) | 67.5 ± 6.7 (0.10) | 59.9 ± 3.2 (0.05) |
| Participant 8 | more affected | 78.4 ± 5.1 (0.07) | 69.1 ± 3.3 (0.05) | 74.0 ± 3.5 (0.05) | 75.1 ± 1.4 (0.02) |
|  | less affected | 79.3 ± 4.8 (0.06) | 69.2 ± 3.5 (0.05) | 74.8 ± 3.0 (0.04) | 75.2 ± 1.4 (0.02) |
| **Step length (cm; mean ± SD (coefficient of variation))** | | | | | |
|  | **Lower limb** | **tSCS-off** | **15 Hz** | **30 Hz** | **50 Hz** |
| Participant 1 | more affected | 24.3 ± 2.0 | 22.2 ± 2.1 | 27.4 ± 1.9 | 29.5 ± 2.2 |
|  | less affected | 32.7 ± 3.5 | 39.1 ± 4.3 | 36.8 ± 2.9 | 34.7 ± 4.9 |
| Participant 2 | more affected | 66.2 ± 4.5 | 65.3 ± 2.2 | 65.5 ± 3.7 | 65.3 ± 2.1 |
|  | less affected | 49.4 ± 6.1 | 54.7 ± 1.5 | 53.1 ± 5.0 | 55.4 ± 2.4 |
| Participant 3 | more affected | 54.8 ± 4.2 | 53.3 ± 5.2 | 54.5 ± 3.9 | 51.3 ± 3.8 |
|  | less affected | 43.1 ± 1.9 | 40.2 ± 3.7 | 40.4 ± 2.5 | 39.2 ± 4.5 |
| Participant 4 | more affected | 54.5 ± 4.5 | 49.6 ± 4.8 | 52.0 ± 4.7 | 51.3 ± 5.2 |
|  | less affected | 44.5 ± 3.4 | 44.8 ± 3.0 | 46.7 ± 2.8 | 46.7 ± 5.4 |
| Participant 5 | more affected | 61.9 ± 4.6 | 59.0 ± 4.7 | 62.2 ± 3.2 | 57.9 ± 6.1 |
|  | less affected | 61.4 ± 1.6 | 60.4 ± 2.2 | 59.4 ± 3.4 | 61.0 ± 3.9 |
| Participant 6 | more affected | 31.4 ± 3.0 | 31.9 ± 3.3 | 30.4 ± 3.3 | 30.0 ± 3.1 |
|  | less affected | 28.2 ± 3.2 | 27.9 ± 2.6 | 26.4 ± 3.8 | 26.4 ± 3.3 |
| Friedman test | more affected | χ^2^(3) = 6.8, p = 0.157 | | | |
|  | less affected | χ^2^(3) = 0.2, p = 1.000 | | | |
| Participant 7 | more affected | 18.9 ± 5.3 (0.28) | 15.6 ± 6.6 (0.42) | 19.9 ± 3.7 (0.19) | 18.9 ± 3.6 (0.19) |
|  | less affected | 20.2 ± 6.1 (0.30) | 17.8 ± 5.1 (0.29) | 11.6 ± 6.4 (0.55) | 17.1 ± 5.1 (0.29) |
| Participant 8 | more affected | 36.4 ± 5.1 (0.14) | 45.3 ± 2.7 (0.06) | 46.4 ± 4.1 (0.09) | 40.4 ± 2.1 (0.05) |
|  | less affected | 29.5 ± 3.1 (0.11) | 38.6 ± 4.0 (0.10) | 35.4 ± 2.0 (0.06) | 32.5 ± 2.5 (0.08) |
| **Stride time (s; mean ± SD (coefficient of variation))** | | | | | |
|  | **Lower limb** | **tSCS-off** | **15 Hz** | **30 Hz** | **50 Hz** |
| Participant 1 | more affected | 4.2 ± 0.6 | 4.1 ± 0.4 | 4.2 ± 0.4 | 4.1 ± 0.4 |
|  | less affected | 4.3 ± 0.5 | 4.2 ± 0.5 | 4.3 ± 0.5 | 4.2 ± 0.5 |
| Participant 2 | more affected | 1.5 ± 0.2 | 1.4 ± 0.1 | 1.4 ± 0.1 | 1.4 ± 0.1 |
|  | less affected | 1.5 ± 0.1 | 1.4 ± 0.1 | 1.4 ± 0.1 | 1.4 ± 0.1 |
| Participant 3 | more affected | 1.4 ± 0.1 | 1.3 ± 0.0 | 1.3 ± 0.0 | 1.3 ± 0.1 |
|  | less affected | 1.4 ± 0.1 | 1.3 ± 0.1 | 1.4 ± 0.1 | 1.3 ± 0.1 |
| Participant 4 | more affected | 2.9 ± 0.2 | 2.6 ± 0.2 | 2.7 ± 0.1 | 2.4 ± 0.1 |
|  | less affected | 2.8 ± 0.1 | 2.6 ± 0.2 | 2.7 ± 0.1 | 2.5 ± 0.1 |
| Participant 5 | more affected | 1.2 ± 0.1 | 1.1 ± 0.0 | 1.1 ± 0.1 | 1.1 ± 0.0 |
|  | less affected | 1.2 ± 0.1 | 1.2 ± 0.0 | 1.1 ± 0.0 | 1.1 ± 0.0 |
| Participant 6 | more affected | 2.2 ± 0.2 | 2.1 ± 0.1 | 2.1 ± 0.1 | 2.3 ± 0.2 |
|  | less affected | 2.2 ± 0.2 | 2.1 ± 0.1 | 2.1 ± 0.1 | 2.3 ± 0.2 |
| Friedman test | more affected | χ^2^(3) = 9.8, p = 0.041 | | | |
|  | less affected | χ^2^(3) = 8.4, p = 0.077 | | | |
| Participant 7 | more affected | 5.0 ± 1.6 (0.31) | 4.9 ± 0.7 (0.15) | 5.3 ± 1.1 (0.20) | 3.7 ± 0.5 (0.13) |
|  | less affected | 5.0 ± 1.5 (0.30) | 4.9 ± 0.7 (0.14) | 5.1 ± 1.0 (0.19) | 3.6 ± 0.4 (0.12) |
| Participant 8 | more affected | 7.4 ± 1.2 (0.16) | 7.1 ± 0.7 (0.10) | 8.0 ± 1.0 (0.12) | 7.6 ± 0.4 (0.05) |
|  | less affected | 7.4 ± 1.1 (0.15) | 7.3 ± 0.8 (0.11) | 8.1 ± 0.9 (0.11) | 7.6 ± 0.4 (0.05) |
| \| **(B) EMG-RMS self-selected speed** \| \| \| --- \| --- \| \| **Rectus femoris RMS during stance phase (µV; mean ± SD)** \| | | | | | |
|  | **Lower limb** | **tSCS-off** | **15 Hz** | **30 Hz** | **50 Hz** |
| Participant 1 | more affected | 48.7 ± 10.5 | 74.3 ± 8.4 | 44.2 ± 7.7 | 55.2 ± 8.7 |
|  | less affected | 73.4 ± 20.7 | 74.1 ± 17.6 | 70.1 ± 17.5 | 65.8 ± 13.6 |
| Participant 2 | more affected | 29.2 ± 7.2 | 29.9 ± 4.6 | 31.2 ± 7.2 | 35.4 ± 7.9 |
|  | less affected | 27.5 ± 5.3 | 25.1 ± 2.2 | 24.2 ± 1.9 | 23.6 ± 3.6 |
| Participant 3 | more affected | 123.7 ± 17.7 | 143.5 ± 17.1 | 144.5 ± 18.4 | 144.1 ± 17.7 |
|  | less affected | 38.2 ± 6.3 | 37.8 ± 6.1 | 39.9 ± 5.9 | 38.3 ± 6.1 |
| Participant 4 | more affected | 27.3 ± 2.0 | 26.0 ± 3.2 | 29.1 ± 7.4 | 31.5 ± 6.6 |
|  | less affected | 29.8 ± 11.6 | 22.3 ± 2.1 | 23.1 ± 2.3 | 25.0 ± 2.8 |
| Participant 5 | more affected | 44.2 ± 10.2 | 46.4 ± 10.8 | 49.4 ± 8.2 | 43.6 ± 3.4 |
|  | less affected | 46.1 ± 2.8 | 45.7 ± 4.2 | 49.5 ± 3.4 | 48.9 ± 6.1 |
| Participant 6 | more affected | 13.6 ± 2.0 | 17.4 ± 1.2 | 15.9 ± 1.7 | 21.0 ± 1.5 |
|  | less affected | 16.4 ± 1.9 | 18.9 ± 1.5 | 17.4 ± 1.4 | 20.6 ± 1.7 |
| Friedman test | more affected | χ^2^(3) = 5.6, p = 0.266 | | | |
|  | less affected | χ^2^(3) = 0.6, p = 1.000 | | | |
| Participant 7 | more affected | 6.7 ± 0.7 | 8.6 ± 0.8 | 9.8 ± 0.7 | 8.7 ± 0.5 |
|  | less affected | 8.0 ± 0.9 | 12.3 ± 1.4 | 10.7 ± 1.2 | 10.9 ± 0.8 |
| Participant 8 | more affected | 21.9 ± 3.1 | 19.3 ± 2.5 | 17.5 ± 1.7 | 21.0 ± 3.7 |
|  | less affected | 39.0 ± 6.1 | 39.8 ± 4.1 | 38.2 ± 4.5 | 37.7 ± 5.6 |
| RMS, Root mean square.  **Rectus femoris RMS during swing phase (µV; mean ± SD)** | | | | | |
|  | **Lower limb** | **tSCS-off** | **15 Hz** | **30 Hz** | **50 Hz** |
| Participant 1 | more affected | 6.1 ± 0.7 | 11.7 ± 1.1 | 6.4 ± 0.7 | 6.9 ± 0.9 |
|  | less affected | 41.3 ± 10.2 | 33.1 ± 20.4 | 40.9 ± 9.2 | 47.8 ± 17.6 |
| Participant 2 | more affected | 30.8 ± 2.2 | 36.6 ± 6.8 | 35.1 ± 3.7 | 33.6 ± 2.6 |
|  | less affected | 13.7 ± 4.8 | 11.9 ± 2.4 | 13.0 ± 3.5 | 12.0 ± 2.4 |
| Participant 3 | more affected | 35.9 ± 15.8 | 41.3 ± 12.1 | 43.8 ± 15.1 | 40.8 ± 9.7 |
|  | less affected | 27.8 ± 10.3 | 31.7 ± 11.4 | 29.6 ± 9.9 | 30.5 ± 17.2 |
| Participant 4 | more affected | 13.0 ± 3.1 | 9.6 ± 1.5 | 10.4 ± 2.4 | 12.8 ± 4.1 |
|  | less affected | 11.9 ±3.0 | 15.8 ± 4.9 | 14.4 ± 5.1 | 14.7 ± 2.1 |
| Participant 5 | more affected | 30.1 ± 11.3 | 31.4 ± 5.2 | 37.2 ± 3.8 | 40.1 ± 9.8 |
|  | less affected | 25.8 ± 7.5 | 29.1 ± 5.3 | 34.3 ± 7.6 | 33.7 ± 3.6 |
| Participant 6 | more affected | 14.3 ± 2.3 | 23.4 ± 2.7 | 20.6 ± 2.7 | 27.2 ± 2.4 |
|  | less affected | 29.4 ± 2.7 | 32.1 ± 4.4 | 28.1 ± 3.4 | 28.6 ± 2.7 |
| Friedman test | more affected | χ^2^(3) = 5.0, p = 0.344 | | | |
|  | less affected | χ^2^(3) = 1.0, p = 1.000 | | | |
| Participant 7 | more affected | 7.0 ± 1.6 | 6.6 ± 2.2 | 7.1 ± 2.1 | 7.0 ± 0.8 |
|  | less affected | 5.4 ± 0.7 | 6.5 ± 1.1 | 7.2 ± 0.7 | 6.9 ± 0.6 |
| Participant 8 | more affected | 41.2 ± 5.5 | 22.2 ± 4.3 | 21.7 ± 2.0 | 30.6 ± 5.2 |
|  | less affected | 50.3 ± 9.1 | 38.6 ± 7.7 | 38.2 ± 5.6 | 37.2 ± 7.4 |
| **Vastus medialis RMS during stance phase (µV; mean ± SD)** | | | | | |
|  | **Lower limb** | **tSCS-off** | **15 Hz** | **30 Hz** | **50 Hz** |
| Participant 1 | more affected | 134.7 ± 32.7 | 178.9 ± 24.1 | 102.1 ± 20.5 | 125.9 ± 23.5 |
|  | less affected | 159.0 ± 42.7 | 128.5 ± 33.1 | 132.5 ± 34.6 | 124.1 ± 23.3 |
| Participant 2 | more affected | 65.6 ± 17.9 | 58.1 ± 8.0 | 56.2 ± 16.8 | 61.9 ± 10.7 |
|  | less affected | 71.5 ± 14.5 | 66.2 ± 6.0 | 62.7 ± 7.6 | 60.8 ± 5.7 |
| Participant 3 | more affected | 198.3 ± 15.6 | 201.2 ± 22.0 | 206.9 ± 25.9 | 191.8 ± 20.1 |
|  | less affected | 57.6 ± 11.6 | 55.9 ± 12.6 | 62.7 ± 16.9 | 57.4 ± 14.1 |
| Participant 4 | more affected | 31.7 ± 3.4 | 34.3 ± 3.3 | 32.7 ± 4.1 | 39.0 ± 7.7 |
|  | less affected | 64.8 ±33.6 | 44.2 ± 6.1 | 46.5 ± 7.4 | 48.5 ± 7.5 |
| Participant 5 | more affected | 162.4 ± 35.2 | 157.7 ± 25.2 | 174.4 ± 23.4 | 139.7 ± 10.8 |
|  | less affected | 39.4 ±3.6 | 39.1 ± 3.9 | 40.9 ± 2.0 | 40.1 ± 4.0 |
| Participant 6 | more affected | 46.8 ± 7.6 | 41.5 ± 4.0 | 33.8 ± 6.0 | 38.0 ± 5.7 |
|  | less affected | 47.1 ± 6.3 | 43.7 ± 3.8 | 42.8 ± 4.3 | 42.4 ± 5.3 |
| Friedman test | more affected | χ^2^(3) = 1.6, p = 1.000 | | | |
|  | less affected | χ^2^(3) = 7.2, p = 0.132 | | | |
| Participant 7 | more affected | 16.2 ± 0.9 | 20.8 ± 1.9 | 20.6 ± 1.6 | 17.5 ± 0.7 |
|  | less affected | 17.5 ± 2.5 | 19.8 ± 3.5 | 16.9 ± 3.0 | 18.7 ± 2.7 |
| Participant 8 | more affected | 25.7 ± 3.6 | 21.8 ± 2.4 | 21.0 ± 2.2 | 22.6 ± 2.1 |
|  | less affected | 35.9 ± 5.4 | 31.9 ± 2.4 | 32.6 ± 5.2 | 35.0 ± 4.0 |
| **Vastus medialis RMS during swing phase (µV; mean ± SD)** | | | | | |
|  | **Lower limb** | **tSCS-off** | **15 Hz** | **30 Hz** | **50 Hz** |
| Participant 1 | more affected | 21.0 ± 6.1 | 67.9 ± 7.4 | 21.3 ± 5.3 | 12.9 ± 2.8 |
|  | less affected | 136.9 ± 39.4 | 94.4 ± 59.9 | 125.6 ± 32.7 | 165.5 ± 57.5 |
| Participant 2 | more affected | 22.5 ± 7.6 | 21.3 ± 5.1 | 22.5 ± 12.1 | 19.6 ± 6.5 |
|  | less affected | 25.2 ± 10.1 | 20.6 ± 5.3 | 25.4 ± 16.5 | 16.4 ± 4.4 |
| Participant 3 | more affected | 15.6 ± 4.1 | 16.2 ± 4.4 | 16.2 ± 4.0 | 18.2 ± 5.8 |
|  | less affected | 16.1 ± 6.4 | 19.0 ± 6.9 | 18.3 ± 8.3 | 17.4 ± 6.7 |
| Participant 4 | more affected | 14.6 ± 3.5 | 10.9 ± 1.5 | 11.1 ± 1.8 | 12.6 ± 2.3 |
|  | less affected | 9.5 ± 1.7 | 11.0 ± 3.5 | 10.2 ± 8.5 | 11.8 ± 3.5 |
| Participant 5 | more affected | 24.2 ± 9.8 | 45.5 ± 14.9 | 69.5 ± 22.3 | 56.9 ± 51.7 |
|  | less affected | 17.0 ± 4.8 | 20.5 ± 4.0 | 30.7 ± 8.6 | 24.0 ± 4.9 |
| Participant 6 | more affected | 21.6 ± 6.9 | 18.3 ± 5.4 | 11.5 ± 4.8 | 18.5 ± 6.4 |
|  | less affected | 17.7 ± 4.8 | 16.0 ± 3.2 | 18.6 ± 6.0 | 14.2 ± 5.3 |
| Friedman test | more affected | χ^2^(3) = 0.6, p = 1.000 | | | |
|  | less affected | χ^2^(3) = 2.6, p = 0.915 | | | |
| Participant 7 | more affected | 12.1 ± 4.2 | 7.8 ± 1.2 | 7.1 ± 2.4 | 10.3 ± 1.4 |
|  | less affected | 4.3 ± 1.2 | 3.9 ± 1.4 | 4.7 ± 2.2 | 3.6 ± 0.8 |
| Participant 8 | more affected | 12.1 ±1.8 | 11.4 ± 0.7 | 13.3 ± 1.0 | 11.5 ± 1.0 |
|  | less affected | 15.4 ± 7.4 | 12.9 ± 2.7 | 12.8 ± 2.5 | 10.4 ± 0.6 |
| **Tibialis anterior RMS during stance phase (µV; mean ± SD)** | | | | | |
|  | **Lower limb** | **tSCS-off** | **15 Hz** | **30 Hz** | **50 Hz** |
| Participant 1 | more affected | 16.0 ± 3.2 | 18.9 ± 3.1 | 14.1 ± 5.3 | 16.3 ± 2.0 |
|  | less affected | 36.4 ± 5.5 | 39.7 ± 10.7 | 39.7 ± 3.8 | 37.7 ± 6.4 |
| Participant 2 | more affected | 49.5 ± 13.4 | 55.9 ± 7.9 | 59.7 ± 10.0 | 49.6 ± 13.2 |
|  | less affected | 57.9 ± 13.7 | 45.0 ± 17.5 | 44.6 ± 12.9 | 39.7 ± 9.6 |
| Participant 3 | more affected | 122.6 ± 62.8 | 89.5 ± 21.4 | 96.6 ± 16.1 | 99.2 ± 22.5 |
|  | less affected | 131.9 ± 14.9 | 134.7 ± 26.2 | 136.0 ± 25.9 | 145.8 ± 21.7 |
| Participant 4 | more affected | 21.3 ± 3.9 | 48.1 ± 3.7 | 44.6 ± 4.0 | 36.7 ± 6.3 |
|  | less affected | 37.1 ± 8.3 | 27.8 ± 6.1 | 31.7 ± 7.2 | 33.8 ± 7.1 |
| Participant 5 | more affected | 39.5 ± 6.2 | 48.3 ± 12.4 | 47.9 ± 7.2 | 52.2 ± 13.0 |
|  | less affected | 59.2 ± 20.1 | 69.8 ± 9.3 | 78.7 ± 11.8 | 68.9 ± 10.6 |
| Participant 6 | more affected | 39.3 ± 11.4 | 40.7 ± 6.4 | 31.4 ± 7.9 | 38.1 ± 14.7 |
|  | less affected | 40.4 ± 6.9 | 38.7 ± 6.4 | 32.3 ± 8.3 | 36.2 ± 10.6 |
| Friedman test | more affected | χ^2^(3) = 3.0, p = 0.783 | | | |
|  | less affected | χ^2^(3) = 0.2, p = 1.000 | | | |
| Participant 7 | more affected | 11.3 ± 2.2 | 11.0 ± 2.7 | 13.2 ± 3.6 | 12.5 ± 2.3 |
|  | less affected | 8.3 ±3.0 | 15.0 ± 5.4 | 14.9 ± 5.0 | 11.4 ± 1.8 |
| Participant 8 | more affected | 16.4 ± 2.3 | 18.9 ± 1.1 | 17.2 ± 0.7 | 16.1 ± 1.3 |
|  | less affected | 10.9 ± 0.8 | 10.9 ± 05 | 10.8 ± 0.4 | 10.6 ± 0.5 |
| **Tibialis anterior RMS during swing phase (µV; mean ± SD)** | | | | | |
|  | **Lower limb** | **tSCS-off** | **15 Hz** | **30 Hz** | **50 Hz** |
| Participant 1 | more affected | 40.4 ± 7.9 | 35.6 ± 7.2 | 35.7 ± 8.5 | 24.9 ± 5.7 |
|  | less affected | 59.9 ± 6.7 | 39.7 ± 7.7 | 52.2 ± 5.8 | 52.6 ± 4.0 |
| Participant 2 | more affected | 53.8 ± 8.2 | 62.8 ± 10.3 | 65.8 ± 9.8 | 59.4 ± 7.7 |
|  | less affected | 50.2 ± 5.6 | 51.2 ± 5.1 | 52.8 ± 8.5 | 49.7 ± 8.4 |
| Participant 3 | more affected | 114.7 ± 25.6 | 111.6 ± 27.5 | 116.6 ± 22.0 | 131.5 ± 21.2 |
|  | less affected | 207.8 ± 68.5 | 230.3 ± 18.9 | 235.6 ± 23.6 | 240.0 ± 24.2 |
| Participant 4 | more affected | 73.8 ± 7.3 | 72.9 ± 7.9 | 72.6 ± 8.5 | 76.7 ± 5.6 |
|  | less affected | 123.8 ± 21.3 | 121.3 ± 17.3 | 124.9 ± 16.5 | 118.0 ± 8.9 |
| Participant 5 | more affected | 84.9 ±14.6 | 83.9 ± 10.5 | 89.3 ± 14.6 | 90.2 ± 11.7 |
|  | less affected | 106.6 ± 10.0 | 116.3 ± 16.2 | 133.3 ± 17.5 | 109.4 ± 14.5 |
| Participant 6 | more affected | 70.6 ± 11.8 | 68.3 ± 7.9 | 58.1 ± 9.3 | 66.5 ± 9.9 |
|  | less affected | 63.8 ± 12.0 | 64.6 ± 5.9 | 60.7 ± 9.7 | 62.7 ± 7.2 |
| Friedman test | more affected | χ^2^(3) = 1.4, p = 1.000 | | | |
|  | less affected | χ^2^(3) = 1.4, p = 1.000 | | | |
| Participant 7 | more affected | 35.2 ± 5.0 | 34.2 ± 5.3 | 45.4 ± 4.2 | 30.9 ± 2.9 |
|  | less affected | 29.2 ± 2.6 | 34.0 ± 4.1 | 32.8 ± 3.2 | 28.8 ± 2.8 |
| Participant 8 | more affected | 40.3 ± 16.4 | 32.5 ± 2.5 | 32.2 ± 3.6 | 41.3 ± 7.7 |
|  | less affected | 64.2 ± 7.5 | 65.2 ± 9.9 | 57.1 ± 4.5 | 67.5 ± 12.8 |
| **Medialis gastrocnemius RMS during stance phase (µV; mean ± SD)** | | | | | |
|  | **Lower limb** | **tSCS-off** | **15 Hz** | **30 Hz** | **50 Hz** |
| Participant 1 | more affected | 64.6 ± 22.6 | 63.0 ± 21.3 | 50.4 ± 22.6 | 51.8 ± 7.4 |
|  | less affected | NT | NT | NT | NT |
| Participant 2 | more affected | 34.4 ± 5.7 | 32.5 ± 2.4 | 33.6 ± 6.2 | 31.3 ± 4.9 |
|  | less affected | 78.3 ± 21.6 | 58.8 ± 7.3 | 64.7 ± 16.0 | 49.2 ± 11.9 |
| Participant 3 | more affected | 8.9 ± 0.8 | 8.7 ± 0.8 | 8.7 ± 0.8 | 8.9 ± 1.0 |
|  | less affected | 53.2 ± 8.2 | 58.0 ± 8.4 | 56.7 ± 10.2 | 61.0 ± 8.0 |
| Participant 4 | more affected | 13.7 ± 0.9 | 52.6 ± 5.6 | 50.9 ± 4.4 | 39.3 ± 5.5 |
|  | less affected | 30.3 ± 7.3 | 30.6 ± 5.1 | 30.7 ± 3.4 | 32.1 ± 4.6 |
| Participant 5 | more affected | 45.7 ± 4.2 | 46.1 ± 4.4 | 56.0 ± 7.1 | 52.4 ± 4.4 |
|  | less affected | 50.2 ± 4.4 | 51.2 ± 3.8 | 63.0 ± 7.8 | 56.8 ± 6.8 |
| Participant 6 | more affected | 41.5 ± 13.2 | 46.0 ± 8.3 | 48.5 ± 13.4 | 44.0 ± 9.3 |
|  | less affected | 32.1 ± 11.6 | 34.9 ± 6.1 | 33.2 ± 9.7 | 34.3 ± 9.3 |
| Friedman test | more affected | χ^2^(3) = 0.8, p = 1.000 | | | |
|  | less affected | χ^2^(3) = 2.4, p = 0.987 | | | |
| Participant 7 | more affected | 7.7 ±1.5 | 9.3 ± 0.6 | 8.5 ± 1.1 | 8.5 ± 0.6 |
|  | less affected | 6.9 ± 1.5 | 5.7 ± 1.2 | 7.4 ± 1.5 | 6.3 ± 0.8 |
| Participant 8 | more affected | 25.6 ± 3.2 | 24.3 ± 1.5 | 24.0 ± 1.5 | 26.0 ± 1.5 |
|  | less affected | 27.1 ± 2.9 | 26.9 ± 3.0 | 27.6 ± 4.2 | 27.7 ± 2.9 |
| **Medialis gastrocnemius RMS during swing phase (µV; mean ± SD)** | | | | | |
|  | **Lower limb** | **tSCS-off** | **15 Hz** | **30 Hz** | **50 Hz** |
| Participant 1 | more affected | 13.5 ± 11.6 | 27.6 ± 15.9 | 21.9 ± 17.5 | 6.8 ± 0.8 |
|  | less affected | NT | NT | NT | NT |
| Participant 2 | more affected | 20.7 ± 5.0 | 20.4 ± 3.5 | 18.7 ± 1.9 | 18.4 ± 4.0 |
|  | less affected | 18.7 ± 6.1 | 16.9 ± 5.3 | 17.3 ± 5.0 | 17.9 ± 6.6 |
| Participant 3 | more affected | 8.5 ± 1.4 | 8.6 ± 1.5 | 8.7 ± 1.2 | 8.8 ± 1.3 |
|  | less affected | 35.7 ± 7.5 | 33.2 ± 8.1 | 30.1 ± 9.0 | 35.9 ± 12.1 |
| Participant 4 | more affected | 7.8 ± 0.5 | 15.8 ± 8.5 | 9.9 ± 4.0 | 9.1 ± 2.0 |
|  | less affected | 8.7 ± 0.8 | 8.1 ± 0.4 | 8.7 ± 3.5 | 8.1 ± 0.5 |
| Participant 5 | more affected | 16.1 ± 4.3 | 15.4 ± 2.3 | 18.4 ± 3.9 | 19.2 ± 3.8 |
|  | less affected | 11.0 ± 5.0 | 8.2 ± 3.5 | 20.1 ± 6.5 | 15.9 ± 7.4 |
| Participant 6 | more affected | 28.7 ± 7.8 | 29.5 ± 4.3 | 27.7 ± 7.2 | 29.1 ± 7.3 |
|  | less affected | 15.7 ± 5.7 | 15.4 ± 4.6 | 14.5 ± 4.5 | 14.5 ± 6.6 |
| Friedman test | more affected | χ^2^(3) = 1.8, p = 1.000 | | | |
|  | less affected | χ^2^(3) = 2.0, p = 1.000 | | | |
| Participant 7 | more affected | 7.8 ± 3.8 | 3.0 ± 0.2 | 4.5 ± 1.6 | 3.1 ± 0.1 |
|  | less affected | 4.5 ± 1.3 | 10.5 ± 11.2 | 9.3 ± 11.4 | 4.4 ± 0.9 |
| Participant 8 | more affected | 11.8 ± 2.0 | 9.8 ± 0.2 | 9.4 ± 0.3 | 10.2 ± 1.1 |
|  | less affected | 15.6 ± 2.0 | 13.3 ± 1.6 | 13.2 ± 3.0 | 15.8 ± 2.8 |
| **Semitendinosus RMS during stance phase (µV; mean ± SD)** | | | | | |
|  |  | **tSCS-off** | **15 Hz** | **30 Hz** | **50 Hz** |
| Participant 1 | more affected | 45.1 ± 4.8 | 42.3 ± 101.5 | 41.0 ± 4.9 | 37.3 ± 6.9 |
|  | less affected | 33.3 ± 6.8 | 28.9 ± 2.7 | 38.4 ± 2.7 | 37.3 ± 2.1 |
| Participant 2 | more affected | 32.0 ± 2.5 | 25.7 ± 0.6 | 28.3 ± 1.0 | 23.8 ± 1.3 |
|  | less affected | 78.8 ± 1.5 | 70.5 ± 5.7 | 70.6 ± 4.1 | 62.7 ± 6.7 |
| Participant 3 | more affected | 52.3 ± 0.8 | 45.7 ± 0.8 | 47.7 ± 0.8 | 44.7 ± 1.0 |
|  | less affected | 17.5 ± 8.2 | 29.7 ± 8.4 | 26.8 ± 10.2 | 20.9 ± 8.0 |
| Participant 4 | more affected | 60.0 ± 7.5 | 61.5 ± 5.4 | 62.7 ± 4.3 | 60.9 ± 5.2 |
|  | less affected | NT | NT | NT | NT |
| Participant 5 | more affected | 31.1 ± 4.2 | 27.8 ± 4.4 | 35.1 ± 7.1 | 30.0 ± 4.4 |
|  | less affected | 56.6 ± 4.4 | 50.2 ± 10.3 | 74.5 ± 7.4 | 55.5 ± 7.3 |
| Participant 6 | more affected | 35.0 ± 6.2 | 35.7 ± 7.4 | 35.4 ± 4.2 | 34.5 ± 12.3 |
|  | less affected | 46.3 ± 12.6 | 42.0 ± 2.3 | 39.2 ± 1.0 | 38.7 ± 0.9 |
| Friedman test | more affected | χ^2^(3) = 7.4, p = 0.120 | | | |
|  | less affected | χ^2^(3) = 3.5, p = 0.647 | | | |
| Participant 7 | more affected | 15.5 ± 11.4 | 6.7 ± 5.0 | 6.4 ± 13.4 | 11.7 ± 11.3 |
|  | less affected | 5.4 ± 25.2 | 5.1 ± 6.9 | 5.5 ± 6.1 | 4.8 ± 4.7 |
| Participant 8 | more affected | 30.2 ± 11.6 | 32.9 ± 18.3 | 32.2 ± 10.3 | 38.8 ± 14.1 |
|  | less affected | 26.0 ± 6.1 | 26.8 ± 5.4 | 27.8 ± 8.9 | 28.4 ± 8.3 |
| **Semitendinosus RMS during swing phase (µV; mean ± SD)** | | | | | |
|  | **Lower limb** | **tSCS-off** | **15 Hz** | **30 Hz** | **50 Hz** |
| Participant 1 | more affected | 34.6 ± 6.2 | 32.6 ± 123.3 | 31.1 ± 5.8 | 21.2 ± 6.6 |
|  | less affected | 13.9 ± 3.3 | 15.9 ± 5.1 | 12.2 ± 6.1 | 14.9 ± 4.0 |
| Participant 2 | more affected | 57.7 ± 0.3 | 63.5 ± 1.8 | 53.9 ± 1.9 | 53.4 ± 1.5 |
|  | less affected | 54.5 ± 5.1 | 50.3 ± 7.3 | 53.5 ± 8.0 | 52.2 ± 6.2 |
| Participant 3 | more affected | 76.0 ± 1.4 | 82.8 ± 1.5 | 81.4 ± 1.2 | 77.1 ± 1.3 |
|  | less affected | 85.4 ± 7.5 | 111.8 ± 8.1 | 106.0 ± 9.0 | 93.2 ± 12.1 |
| Participant 4 | more affected | 37.3 ± 4.9 | 35.0 ± 9.5 | 37.2 ± 5.7 | 38.2 ± 7.5 |
|  | less affected | NT | NT | NT | NT |
| Participant 5 | more affected | 38.7 ± 4.3 | 36.4 ± 2.3 | 38.1 ± 3.9 | 38.4 ± 3.8 |
|  | less affected | 53.0 ± 8.3 | 55.3 ± 12.1 | 63.2 ± 14.2 | 56.9 ± 14.2 |
| Participant 6 | more affected | 29.8 ± 7.1 | 25.9 ± 9.4 | 28.5 ± 6.5 | 29.0 ± 6.0 |
|  | less affected | 36.3 ± 1.9 | 38.0 ± 2.5 | 35.5 ± 3.2 | 34.2 ± 2.8 |
| Friedman test | more affected | χ^2^(3) = 2.2, p = 1.000 | | | |
|  | less affected | χ^2^(3) = 1.3, p = 1.000 | | | |
| Participant 7 | more affected | 28.4 ± 5.6 | 16.6 ± 21.4 | 14.9 ± 22.7 | 21.9 ± 25.7 |
|  | less affected | 10.0 ± 2.6 | 9.1 ± 3.4 | 10.6 ± 5.4 | 9.6 ± 6.7 |
| Participant 8 | more affected | 46.3 ± 13.9 | 27.6 ± 8.1 | 26.6 ± 8.5 | 36.7 ± 6.6 |
|  | less affected | 46.9 ± 4.7 | 32.4 ± 6.0 | 33.7 ± 8.6 | 39.5 ± 10.0 |
